# A Gβ protein and the TupA Co-Regulator Bind to Protein Kinase A Tpk2 to Act as Antagonistic Molecular Switches of Fungal Morphological Changes

**DOI:** 10.1371/journal.pone.0136866

**Published:** 2015-09-03

**Authors:** Thamarai K. Janganan, Gongyou Chen, Daliang Chen, João F. Menino, Fernando Rodrigues, Maria I. Borges-Walmsley, Adrian R. Walmsley

**Affiliations:** 1 School of Biological and Biomedical Sciences, Durham University, Durham, United Kingdom; 2 Life and Health Sciences Research Institute (ICVS), School of Health Sciences, University of Minho, Braga, Portugal; 3 ICVS/3B’s - PT Government Associate Laboratory, University of Minho, Braga/Guimarães, Portugal; Universidade de Sao Paulo, BRAZIL

## Abstract

The human pathogenic fungus *Paracoccidioides brasiliensis (Pb)* undergoes a morphological transition from a saprobic mycelium to pathogenic yeast that is controlled by the cAMP-signaling pathway. There is a change in the expression of the Gβ-protein PbGpb1, which interacts with adenylate cyclase, during this morphological transition. We exploited the fact that the cAMP-signaling pathway of *Saccharomyces cerevisiae* does not include a Gβ-protein to probe the functional role of PbGpb1. We present data that indicates that PbGpb1 and the transcriptional regulator PbTupA both bind to the PKA protein PbTpk2. *PbTPK2* was able to complement a *TPK2Δ* strain of *S*. *cerevisiae*, *XPY5a/α*, which was defective in pseudohyphal growth. Whilst *PbGPB1* had no effect on the parent *S*. *cerevisiae* strain, *MLY61a/α*, it repressed the filamentous growth of *XPY5a/α* transformed with *PbTPK2*, behaviour that correlated with a reduced expression of the floculin *FLO11*. *In vitro*, PbGpb1 reduced the kinase activity of PbTpk2, suggesting that inhibition of PbTpk2 by PbGpb1 reduces the level of expression of Flo11, antagonizing the filamentous growth of the cells. In contrast, expressing the co-regulator *PbTUPA* in *XPY5a/α* cells transformed with *PbTPK2*, but not untransformed cells, induced hyperfilamentous growth, which could be antagonized by co-transforming the cells with *PbGPB1*. *PbTUPA* was unable to induce the hyperfilamentous growth of a *FLO8Δ* strain, suggesting that PbTupA functions in conjunction with the transcription factor Flo8 to control Flo11 expression. Our data indicates that *P*. *brasiliensis* PbGpb1 and PbTupA, both of which have WD/β-propeller structures, bind to PbTpk2 to act as antagonistic molecular switches of cell morphology, with PbTupA and PbGpb1 inducing and repressing filamentous growth, respectively. Our findings define a potential mechanism for controlling the morphological switch that underpins the virulence of dimorphic fungi.

## Introduction

*Paracoccidioides brasiliensis* is one of a group of six phylogenetically related ascomycete fungi that, from more than a hundred-thousand fungi in the environment, have adapted for survival in mammalian hosts [1–3]. These six fungi are dimorphic, undergoing extensive changes that allow them to switch from a nonpathogenic filamentous mycelium, usually found in soil, to pathogenic single-cellular yeast that every year causes infections in millions of people across the globe. Infection is the result of hypha-fragments or spores released from mycelium, which are inhaled by the host, exposing them to an increased temperature that triggers the morphological switch. The pathogenicity of these fungi is intimately linked to the morphological change since strains that are unable to transform from mycelium to yeast are often avirulent [3,4]. Our knowledge of the mechanisms that these fungi use to sense and respond to the temperature change to switch morphology is still rudimentary.

The cAMP-signaling pathway has been shown to be important in controlling morphological changes and the pathogenicity of several animal and plant pathogenic fungi [1–5], including the plant pathogens *Ustilago maydis* [6,7] and *Magnaporthe grisea* [8,9] and the human pathogens *Aspergillus fumigatus* [10,11], *Candida albicans* [12,13] and *Cryptococcus neoformans* [14,15]. Furthermore, the importance of the cAMP-signaling pathway in controlling the morphological switch in *Histoplasma capsulatum* [16,17] and *P*. *brasiliensis* has [18,19] has been established. One of the best-studied fungal c-AMP-signaling pathways that control morphological changes is that in *Saccharomyces cerevisiae*, which produces pseudohyphae in response to nitrogen limitation in the presence of a rapidly fermentable sugar [20,21].

In *S*. *cerevisiae*, the synthesis of cAMP from ATP is catalyzed by a single adenylate cyclase, Cyr1 [22]; whilst the degradation of cAMP is catalyzed by low- and high-affinity phosphodiesterases, Pde1 and Pde2, respectively [23,24]. Stimulation of adenylate cyclase by the Gα-protein Gpa2, which is coupled to the Gpr1 receptor [15,25] that can be activated by extracellular sucrose and glucose [26], is required for pseudohyphal differentiation [27]. The RGS protein Rgs2 stimulates the intrinsic GTPase activity on Gpa2 to attenuate its action [28]. In contrast to other Gα-proteins, Gpa2 does not associate with canonical Gβγ subunits [29,30], but instead binds the two kelch-repeat proteins, Krh1 and Krh2, which appear to act as Gβ mimics [31,32]. When glucose is added to glucose-deprived cells, cAMP rapidly and transiently accumulates and triggers the activation of Protein Kinase A (PKA) [33]. PKA is an inactive tetramer composed of two regulatory subunits, each encoded by the *BCY1* gene, and two catalytic subunits, encoded by the *TPK1*, *TPK2* or *TPK3* gene, in the absence of cAMP [34]; but upon binding of cAMP to the regulatory subunits, the partially redundant catalytic subunits, dissociate and become active [35,36]. Activated PKA subsequently phosphorylates protein kinases, transcription factors, and other substrates to control various physiological processes. Recent studies have shown that the Tpk proteins bind Krh1, which apparently stimulating their association with Bcy1 to attenuate their activity [37,38]. Mutants with constitutively high PKA activity are hyperfilamentous; whereas those with low PKA activity cannot switch to the filamentous form [20,39]. Several phenotypes are regulated differently by PKA isoforms: for example, Tpk2 stimulates pseudohyphal morphogenesis, whereas Tpk1 and Tpk3 have a repressing effect [20,40]. Down-stream targets of Tpk2 include the transcription factors Flo8, required for the expression of Flo11, a glycerol-phosphoinositol—anchored cell surface protein [20,41] that promotes mother-daughter cell adhesion required for pseudohyphal growth [42], as well as Sfl1 [40], proposed to inhibit pseudohyphal growth by recruitment of the Ssn6–Tup1 co-repressor complex [43]. Flo8 and Sfl1 antagonistically control expression of *FLO11* via a common promoter element: Tpk2 phosphorylates Flo8 activating its binding to the *FLO11* promoter, whilst it phosphorylates Sfl1 to inhibit its binding to the *FLO11* promoter [44].

Recent studies have revealed that *C*. *albicans* has homologs of Flo8 [45] and Sfl1 [46,47] that have analogous roles in controlling hyphal development. Although *C*. *albicans* has homologs of Tup1 [48,49] and Ssn6 [50,51], which can repress filamentous growth, these proteins have not, to date, been shown to interact with Flo8 and Sfl1. However, Tup1 appears to act in conjunction with the Nrg1 [52,53] and Rfg1 [54,55] repressors of filamentous growth. In contrast to the constitutive filamentation of a *tup1Δ* strain of *C*. *albicans*; a *tupAΔ* strain of the dimorphic fungus *P*. *marneffei* had reduced filamentation [56]. *P*. *marneffei* TupA is proposed to promote filamentous growth, whilst repressing spore and yeast development [56].

Previously we established that the morphology of *P*. *brasiliensis* is influenced by the addition of cAMP, indicating that the cAMP-signaling pathway is important in controlling the morphological transition from mycelium to yeast [19]. Importantly, we found that there is a change in the expression of the Gβ-protein Gpb1 during the morphological transition and that it interacts with the N-terminal domain of AC; suggestive of a novel signaling mechanism in which the activity of AC is differentially modulated by Gpa1 and Gpb1, such that a transient increase in Gpb1 during the morphological transition is used to attenuate cAMP-levels [19]. Similarly, Asc1, a 7 WD-domain Gβ-mimic, has been shown to antagonize the function of Gpa2 in *S*. *cerevisiae*, not only by binding Gpa2 but also Cyr1 to decrease cAMP-levels [57]. However, in apparent contradiction to its negative role on cAMP-signaling, an *asc1Δ*/*asc1Δ* diploid strain had reduced *FLO11* transcript levels and did not form pseudohyphae in response to nitrogen starvation [58]. One possibility is that Asc1 works differentially through both AC and PKA.

Progress in analyzing the role of PbGpb1 in *P*. *brasiliensis* has been hindered by the fact that molecular genetic techniques are only just emerging [59–61]. We have transformed the *PbGPB1* gene into *S*. *cerevisiae* in an attempt to further our understanding of its function. Our rationale in doing so was to exploit the fact that no true Gβ-protein has been found to function in the *S*. *cerevisiae* cAMP-signaling pathway and as a result its components would not be expected to interact with PbGpb1; for PbGpb1 to exert an effect on *S*. *cerevisiae* it would be necessary to replace components of the *S*. *cerevisiae* cAMP-signaling pathway with those from *P*. *brasiliensis* with which PbGpb1 interacts. Using this approach we present, herein, data that indicates that PbGpb1 and the co-regulator PbTupA from *P*. *brasiliensis* both bind to PbTpk2 to act as molecular switches of cell morphology: PbTupA promotes filamentous growth, whilst PbGpb1 acts antagonistically to repress filamentous growth, in *S*. *cerevisiae*. The controlled differential expression of these genes would provide a mechanism for switching the morphology, between the mycelia and yeast forms, which underpins the virulence of many dimorphic fungi that possess homologues of PbGpb1 and PbTupA.

## Materials and Methods

### Strains and plasmids

The strains used for this investigation are described in S1 Table. The plasmids and the primers used for plasmid constructions used in this investigation are described in S2 and S3 Tables, respectively.

### Construction of cDNA libraries

The construction of the *P*. *brasiliensis* yeast cDNA library in the vector pDNR-LIB has been reported previously [19]. The *PbTPK2* and *PbTUPA* genes were cloned from the Creator pDNR library using the primers given in S4 Table. A cDNA library was constructed in the yeast two-hybrid prey vector pGADT7 (Clontech, CA, USA) by transferring cDNA fragments from our pDNR library to this vector.

### Green and red fluorescent protein expression in yeast

The *GFP* gene (derived from pEGFP-Actin (Clontech)) was cloned into the *EcoR*Ι and *Sal*Ι sites of p426MET25 [62], whilst the *mRFP* gene (derived from pVT100U-mRFP) was cloned into the *Hind*III and *Sal*I sites of p426MET25, to place their expression under the *met25* constitutive promoter and *cyc* transcriptional terminator, and the *S*. *cerevisiae* MLY61a/α strain was transformed with these vectors. Yeast cells were grown on SD minus uracil broth at 30°C for 4–5 hours and GFP or mRFP expression verified by fluorescence microscopy by observing the cells (smeared on a microscopy slide, air dried and fixed with 4% formaldehyde for 20 minutes) under oil immersion at 100x magnification using an Olympus 1X71 microscope. Subsequently, 2–3 day old colonies (grown on SD minus uracil plates at 30°C) were screened for GFP and/or mRFP fluorescence using a Leica M165 FC stereomicroscope with green and red filters. Generally, to select for co-transformants expressing both GFP and mRFP fusion proteins, we initially transformed the yeast cells with the GFP construct and selected for transformants on the basis of their green fluorescence, and then we used these cells for transformation with the mRFP construct, selecting the co-transformants on the basis of their red fluorescence.

### Microscopy

For the microscopic assays of *S*. *cerevisiae* cells for pseudohyphal growth, cells were streaked on SLAD (Synthetic Low-Ammonium Dextrose) medium (0.17% yeast nitrogen base, 2% dextrose, 50 or 200 μM ammonium sulfate, 2% Agar) [27] and incubated at 30°C for 3 to 6 days. Individual colonies were observed under a Nickon Eclipse E400 microscope at 20x magnification. To test for invasive growth of *S*. *cerevisiae* transformants, expressing GFP and mRFP fusion proteins, these were grown on SD minus uracil plates at 30°C for 3 to 6 days and pictured using a Leica M165 FC stereomicroscope, with green and red filters. Invasive growth was identified as a dark elliptical shape within the green or red colony. If agar-invasive growth was apparent, then the surface colony was removed by washing with PBS (phosphate buffered saline) and the colony re-screened by fluorescence microscopy for the dark elliptical indentation.

Confocal microscopy was used to observe *S*. *cerevisiae* cells expressing GFP and mRFP. The nuclei of yeast cells (2 days old) transformed with GFP and mRFP constructs were analyzed by DAPI staining by smearing a colony on a microscopy slide with saline, air dried and fixed with 4% formaldehyde for 20 minutes. The slide was washed three-times with PBS and a drop of mounting medium with DAPI solution (Vectashield mounting medium H-1200) was placed over the slide, covered with a cover slip and left at room temperature for 30 minutes to dry and observed under a Zeiss LSM 510 META confocal microscope with 40x oil immersion objective. LSM 5 image browser and Image J software was used to process all of the pictures.

### Complementation of *S*. *cerevisiae* SGY446 and XPY5a/α PKA-mutants

To enable the initial selection of *S*. *cerevisiae* cells transformed with the *P*. *brasiliensis TPK2* and *TPK1* genes, constructs were made for the expression of the proteins fused with N-terminal GFP: *PbTPK2* ^*(1–225)*^, *PbTPK2* ^*(226–583)*^ and *PbTPK1* ^*(135–560)*^ were ligated into the *BamHІ*/*EcoRІ* sites of p426-MET25-GFP. The latter two constructs incorporated the catalytic domains of these kinases. The *S*. *cerevisiae* haploid strain SGY446 (MATα *tpk1Δ*::ADE8 *tpk2-63(Ts) tpk3*::TRP1 BCY1 *ura3-52 his3 leu2-3*,*112 trp1 ade8*; haploid stran that is defective in growth at 37°C and used for PKA complementation assays) [63] was transformed with these vectors and the transformants selected for the uracil marker on SD minus uracil plates, grown at 25°C for 5 to 6 days. The transformants were replica plated on SD minus uracil and incubated at 25°C and 37°C. *PbTPK2* complementation was detected as growth at 37°C (but cells expressing *PbTPK1* could not grow at 37°C). The same constructs were also transformed in to *S*. *cerevisiae* diploid strain XPY5a/α (*Δtpk2*::G418/ *Δtpk2*::G418 ura3-52/ ura3-52 MATa/α; diploid strain defective in pseudohyphal growth and used for PKA complementation assays) [20] and selected on SD minus uracil plates, grown at 30°C for 5 to 6 days. For the pseudohyphal analyses, the transformants were streaked on SLAD medium and incubated at 30°C for 6 days. Single colonies on the SLAD agar plate were observed by light microscopy at 20x magnification (using a Nickon Eclipse E400 microscope). Subsequently, a full length *PbTPK2* construct was made, by ligating *PbTPK2* ^*(1–583)*^ into the *BamHІ*/*EcoRІ* sites of p426-MET25, for the expression of PbTpk2 without GFP: this construct complemented the growth phenotypes of both *S*. *cerevisiae* SGY446 and XPY5a/α.

### Site-directed mutagenesis

The PbTpk2 K301R and Pb-Gpb1 S109R and S151R derivatives were constructed by site-directed mutagenesis of the *PbTPK2* and *PbGPB1* genes using the QuickChange XL Site-Directed Mutagenesis kit (Stratagene) using the p426MET25 Tpk2/Gpb1 constructs as the template, with the primers given is S3 Table. PCR reactions were performed using the following cycling parameters: initial activation 95°C—1 min, denaturation 95°C—50 sec, annealing 55°C—50 sec and extension 68°C—9 min for 17 cycles, with a final extension at 68°C for 10 mins. After PCR, the template was digested with *Dpn*1 for 1 hour and 5μl PCR mix transformed into XL1 blue and colonies selected for ampicillin resistance. Plasmids were extracted from the colonies and their DNA sequenced.

### RNA extraction and real time PCR for *FLO11* transcript analysis

Yeast cells were grown in SLAD broth, supplemented with 50μM ammonium sulphate, until an OD_600_ of 0.6–0.7 had been reached, harvested and the total RNA was extracted using Ribopure ^TM^ (Ambion, Applied biosystem) according to the manufactures’ protocol. Then 0.5μg of total RNA was used for RT Q PCR using a Rotorgene Q PCR instrument (QIAGEN). The LightCycler^(TM)^ RNA master SYBR green І (Roche Applied Science) was used with a reaction mixture of 8.2μl of RNase free water, 1.3μl of manganese acetate, 7.5μl of RNA master mix, 1μl of (0.5μg/μl) RNA and 2μl of primers; *FLO11* or *actin* (5μM of forward and reverse) primer mix. The RT PCR was performed with the following cycling parameters: 61°C for 20 minutes for reverse transcription, followed by initial activation 95°C for 30 seconds, initial denaturation 95°C for 5 seconds, annealing 55°C for 15 seconds and extension 72°C for 12 seconds, for 45 cycles (see S3 Table for primers). The *FLO11* expression was quantified relative to the *Actin* value. The results were analysed using the Rotor-gene software 6.1.93. The experiments were performed in triplicates.

### Yeast two-hybrid analysis and screening

The Matchmaker Two-Hybrid System 3 (Clontech) was used to test for protein-protein interactions and to screen libraries for potential interacting protein partners. To test for specific interactions, the genes to be used as bait and prey, respectively, were sub-cloned into the vectors pGBKT7 and pGADT7 for use in yeast two-hybrid screens, whilst for library screening the bait gene was ligated into pGBKT7 for screening our pGADT7 cDNA library. Generally, the genes were swapped between the plasmids to confirm interactions. To test for specific interactions, co-transformants were generated by introducing both bait and prey vectors into yeast strain AH109 by co-transformation. For library screening, the bait vector was introduced into AH109 first, followed by sequential cotransformation with 20 μg of the prey library plasmids. Experimental procedures were conducted in accord with the Matchmaker GAL4 Two-Hybrid System 3 manual and the Yeast Protocol Handbook (Clontech). Protein interactions were initially identified by observing the growth of transformants on SD-Ade/-His/-Leu/-Trp plates and confirmed as growth of blue cultures on plates supplemented with α-X-gal. Some of the two-hybrid interactions were further confirmed using a quantitative β-galactosidase assay with *o*-nitro-phenyl-β-pyronaside (ONPG) as the substrate. After library screening, candidate transformants were twice re-streaked on SD-Ade/-His/-Leu/-Trp plates to allow loss of non-interacting prey vectors. Interacting prey vectors were purified using a Yeast Plasmid Isolation kit (Clontech) and subjected to DNA sequence analysis to confirm the identities of the interacting gene products.

### Protein expression in *E*. *coli*

The *PbTPK2* ^*(226–583)*^ and *PbTUPA* genes from *P*. *brasiliensis* were cloned into pET21d(+) for expression of the His_6_-tagged proteins in *E*. *coli* BL21(DE3) PlysS (Stratagene). 10 ml of cells, grown for 3–4 hours, was used as a starter culture for 1L of LB medium. Cells were induced at an OD_600_ of 1.1 for PbTpk2 and 0.5 for PbTupA with 0.1 mM IPTG for 5 hours at 20°C in LB. The cells were harvested, resuspended in buffer A (20 mM Tris HCl, pH 8.0, 500 mM NaCl, 1% Triton X-100, 1 mM β-mercapto ethanol, 10 mM imidazole, 1 mM DTT, 10% glycerol) and DNase-I (1000 units/ml) and incubated on ice for 15 minutes. The cells were then passed twice through a Constant Systems (UK) cell disruptor (run at 15Kpsi) to lyse the cells, which were harvested by centrifugation at 43,000 rpm for an hour. The supernatant was mixed with nickel-sepharose beads (Qiagen) and incubated at 4°C in an end-over-rotator for 30 minutes. The beads were packed into a column and washed 3 times with buffer A, supplemented with 25, 50 and 75 mM imidazole, and the proteins eluted with buffer A, supplemented with a 100–500 mM imidazole gradient. The elution fractions were run on 4 to 12% SDS (NuPAGE precast) polyacrylamide gels and the purified proteins confirmed by Western-blotting. The protein concentrations were measure a BCA^TM^ protein assay (Pierce).

The *PbCYR1* ^*453-678*^, *PbGPB1*, *PbTPK2* ^(1–225)^ and *PbTPK2* ^(1–583)^ full length genes were cloned into pGEX6p-3 (GE Healthcare), to enable expression of the glutathione S-transferease fusion-proteins, and these vectors used to transformed *E*. *coli* BL21(DE3) codon plus (Stratagene) cells, which were grown in 2YT, at 25°C with shaking at 200 rpm, before induction with 0.1 mM IPTG. Cells were harvested by centrifugation, resuspended and disrupted by passage through a Constant Systems cell disruptor; 0.1% Triton X-100 was added to the disrupted cells and the debris collected by ultracentrifugation (43,000 rpm, 1hr). The supernatant was mixed with GST-beads and incubated on a rotator for 30 minutes at 4°C, loaded into a glass column and washed with PBS and finally with GST elution buffer (50mM Tris/HCl pH 8.0, 10 mM glutothione). The elution fractions were run on 4 to 12% SDS-PAGE (Nu PAGE precast) polyacrylamide gels. The protein concentrations were measured using a BCA^TM^ protein assay kit (Pierce).

### Pulldown assays

GST pulldown assays were performed with GST fusion proteins as bait and His-tagged proteins as prey. PbCyr1^453-678^-GST, PbGpb1-GST, PbTpk2^(1–225)^-GST and GST were dialyzed in PBS and incubated at 4°C for 40 minutes with 40 μl of GST beads to immobilize the proteins. The GST beads were washed 5x with PBS and then mixed with 1 ml of 2.5 mg/ml PbTpk2^(226–583)^ (or 0.4 ml 2.0 mg/ml PbTupA) (dialyzed with pulldown buffer: 20mM HEPES pH 7.9, 600 mM NaCl, 0.05% Tween 20, 5% glycerol and 1mM DTT). The beads were incubated overnight (12 hrs) on an end-over-rotator at 4°C and then washed 9x with 1ml of pull-down buffer. 30 μl of 4x NuPAGE LDS sample buffer (Invitrogen) was mixed with the beads, which were boiled for 5 minutes. After a brief centrifugation, 20 μl of elutant was loaded on a 12% NuPAGE SDS precast gel. The proteins on the gel were transferred to a PVDF membrane and PbTpk2 (or PbTupA) was detected by anti-hexahistidine monoclonal antibodies (Sigma).

### Western-blots

Antibodies were to the His-tag (Sigma), GFP (Clontech) or commercially produced polyclonal antibodies (Invitrogen) raised in rabbits to the following specific oligopeptides: PbGpb1—CDIRADRELNTYQSD or PbTpk2 –SQFDRYPEETEPYG were used. Proteins were separated by SDS-PAGE (on 4 to 12% polyacrylamide gels) and electrotransferred to PVDF membrane. Blots were incubated with the respective antibodies (e.g anti-His_6_ at 1:5000 dilution; anti-GFP at 1:100 dilution and PbGpb1 and PbTpk2 specific antibodies at 1:2500 dilution). Alkaline-phosphatase-conjugated goat anti-mouse IgG (1:2500 dilution) and horse radish peroxidase-conjugated anti-rabbit IgG (1:2500 dilution) were used as secondary antibodies for His_6_, GFP, mRFP and specific-protein blots, respectively. Western-blots were used routinely to confirm the presence of bait and prey proteins.

### PKA kinase assay

The kinase activity of PKA was determined using an ADP-Glo^TM^ kinase assay kit (Promega) according to the manufactures instructions. Prior to the assay PbTpk2 and PbGpb1 were dialyzed (using Slide-A-Lyzer^®^ 10k Dialysis Cassette) against the kinase reaction buffer (KRB: 40mM Tris-HCL (pH 7.5), 20 mM MgCl_2_), supplemented with 100mM NaCl and 1mM DTT. The assay reaction mix (100 μl, in the well of a 96-well plate) consisted of 3 μM PbTpk2 and 200 μM ATP in KRB, to which was added either 50 μM Kemptide and/or 4 μM PbGpb1. The reaction mix was incubated at room temperature for 1 hour, 25 μl removed and mixed with 25 μl of ADP-Glo reagent, incubated for a further 40 minutes, before adding 50 μl of kinase detection reagent, followed by a further incubation of 40 minutes, after which the luminescence of the samples was determined. A calibration curve was constructed luminescence of a series of [ATP]/[ADP] ratios; from which the % ATP hydrolysis for the PKA samples could be determined. The kinase activity was normalised relative to that of the positive control, consisting of 3 μM PbTpk2, 200 μM ATP and 50 μM Kemptide in KRB. Measurements were made in triplicate.

### Ethics statement

This project did not include any work with live animals for which ethical approval is required. The production of rabbit antibodies was carried out by Invitrogen in the UK.

### Nucleotide sequence accession number

The GenBank accession numbers for the *P*. *brasiliensis* genes used in this study are as follows: *CYR1* (AAS01025), *GPA1* (AAT40562), *GPA2* (AAT40564), *GPA3* (AAT40563), *GPB1* (AAT40565), *GPG1* (ABS18965), *TPK2* (PAAG_00108.1) and *TUPA* (PAAG_03649.1).

## Results

### Identification of *P*. *brasiliensis* protein kinase A

In order to extend our analysis of the cAMP-signaling-pathway in *P*. *brasiliensis* we initially sought to identify the down-stream target of AC, protein kinase A (PKA). We cloned a single *TPK* gene (*PbTPK2*; PAAG_00108.1) that encodes the catalytic subunit of PKA, which had most sequence identity to PkaC1 (XP75552) (S1 Fig), which regulates the development and virulence of *A*. *fumigatus* [10,64]. The genome sequence for *A*. *fumigatus* [65] indicates that there is a second Pka (XP753780); causing us to screen the *P*. *brasiliensis* genome sequence, identifying PbTpk1 (PADG_07326.1), which had 27.3% sequence identity with ScTpk2 (S1 Fig). In common with other fungal Tpk proteins, both *P*. *brasilensis* Tpk proteins had extensive N-terminal domains that are not present in the catalytic subunits of mammalian PKA (S2 Fig).

### *PbTPK2* can complement a *TPK2* *Δ* *S*. *cerevisiae*

**Fig 1:**
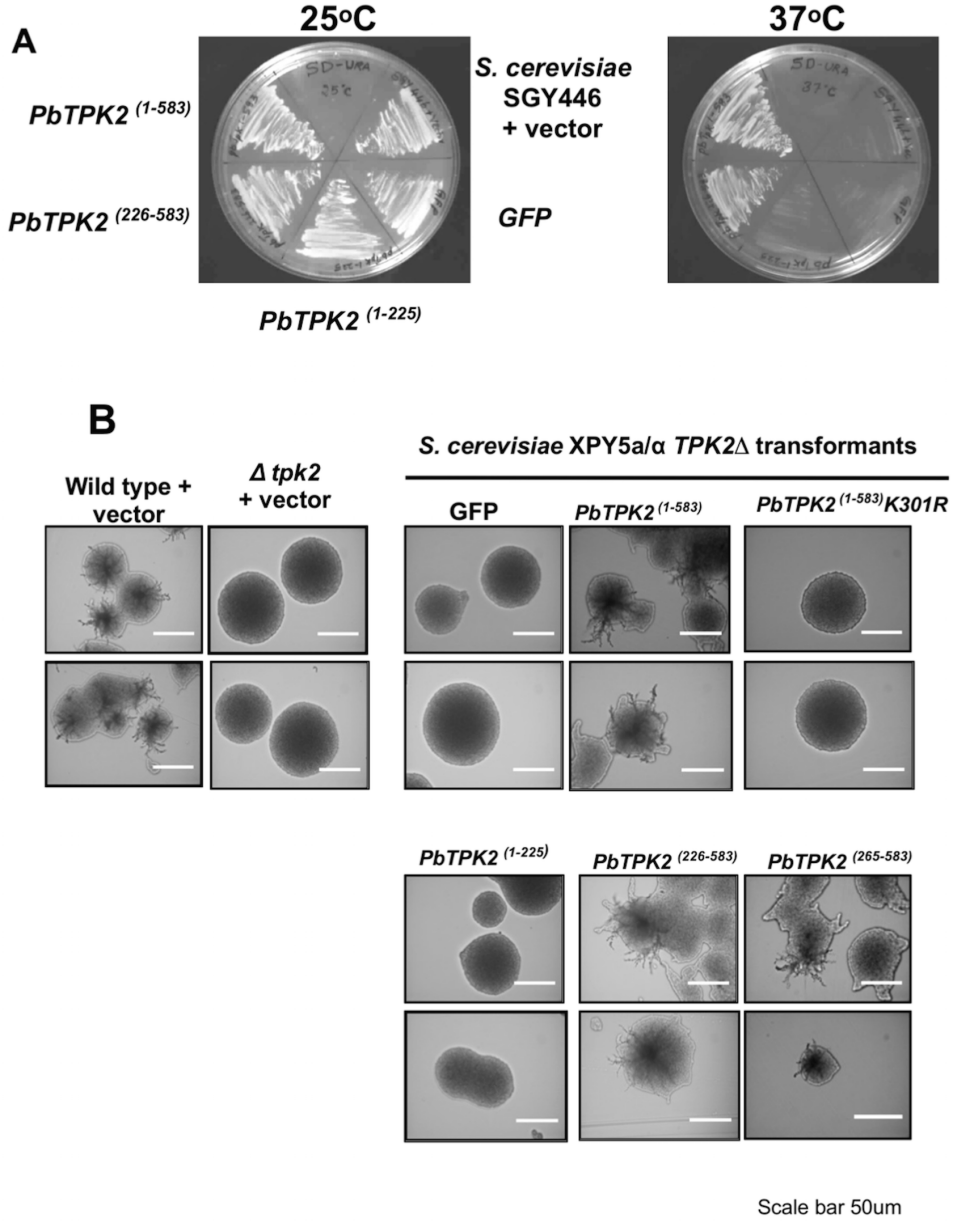
**(A) *P*. *brasiliensis TPK2* complements the growth defect of the *S*. *cerevisiae ΔTPK2* Ts mutant strain SGY446.** The *S*. *cerevisiae* haploid strain SGY446 that cannot grow at 37°C was transformed with p426MET25 (a), p426MET25-GFP (b), and the p426MET25-GFP constructs for the expression of GFP-fusion proteins of the N-terminal domain (PbTpk2^(1–225)^) (c), C-terminal domain (PbTpk2^(226–583)^) (d)) and full-length (PbTpk2^(1–583)^) PbTpk2 (e) from *P*. *brasiliensis* and incubated at 25°C and 37°C. The cells expressing the C-terminal domain (d) and full-length PbTpk2 (e) were able to grow at 37°C. **(B) *P*. *brasiliensis PbTPK2* complements the defect in the ability of the *S*. *cerevisiae ΔTPK2* XPY5a/α strain to form pseudohyphae.** The *S*. *cerevisiae* diploid strain XPY5a/α was transformed with constructs for the expression of GFP (GFP), the the N-terminal domain (PbTpk2^(1–225)^), C-terminal domain (PbTpk2^(226–583)^ and PbTpk2^(265–583)^), full-length (PbTpk2^(1–583)^) PbTpk2 and the K301R full-length PbTpk2 derivative from *P*. *brasiliensis* and the transformants were analysed for pseudohyphal growth in SLAD agar, containing 50 μM (upper panel) or 200 μM (lower panel) ammonium sulfate. Single colonies from the agar plate were observed at 20x magnification in an Eclipse E-400 microscope. The scale bar is 50 μm. The cells expressing the C-terminal domains and full-length PbTpk2 were able to form pseudohyphae, but cells expressing the full-length K301R PbTpk2 derivative, defective in kinase activity, were unable to form pseudohyphae.

We sought to test whether the *PbTPK2* gene could complement a *TPK2*-deletion in the haploid strain SGY446 [63], which is temperature sensitive and unable to grow at 37°C, and the diploid strain XPY5a/α [20], which is unable to produce pseudohypha under low nitrogen conditions. Initially we used a *PbTPK2-GFP* fusion construct to screen for *S*. *cerevisiae* transformant colonies that produced green fluorescence; these transformants demonstrate that the *P*. *brasiliensis TPK2* gene complemented the SGY446 and XPY5a/α phenotypes, to grow at 37°C (Fig 1A), and to produce pseudohyphae (Fig 1B), respectively. Subsequently, we used these phenotypes to select *P*. *brasiliensis TPK2* transformants directly to confirm the complementation. In order to confirm that the ability of *P*. *brasiliensis TPK2* to complemented the SGY446 and XPY5a/α phenotypes was attributable to its kinase activity, rather than indirectly by sequestering the ScBcy1 regulatory proteins to release ScTpk1 and 3 in the *S*. *cerevisiae* cells, a mutation (e.g. K301R) was introduced into *PbTPK2* to render the protein derivative kinase inactive [44]. Although both the wild-type and K301R derivative of PbTpk2 interacted with ScBcy1 in yeast two-hybrid (Y2H) assays (data not shown), the K301R derivative was unable to complement the SGY446 and XPY5a/α cells to grow at 37°C (data not shown for SGY446 complementation) and produce pseudohyphae (Fig 1B), respectively. We made three additional constructs to express the N- and C-terminal domains of PbTpk2, PbTpk2^(1–225)^, PbTpk2^(226–583)^ and PbTpk2^(265–583)^ respectively, and tested these for complementation of SGY446 and XPY5a/α; PbTpk^(226–583)^ and PbTpk2^(265–583)^ (data not shown for SGY446 complementation) was able to complement both strains but PbTpk2^(1–225)^ could not (Fig 1A and 1B), indicating that the C-terminal domain is sufficient for functionality and for enabling filamentous growth. Similarly, we tested if PbTpk1 could complement the *S*. *cerevisiae* SGY446 and XPY5a/α mutants but we found that, in contrast to PbTpk2, it was unable to support the growth of SGY446 at 37°C (data not shown for SGY446 complementation) and did not enable the production of pseudohyphae by XPY5a/α (S3A Fig), suggesting PbTpk1 does not function as part of the pathway that controls filamentation.

### PbTpk2 interacts with both G proteins and AC

**Fig 2:**
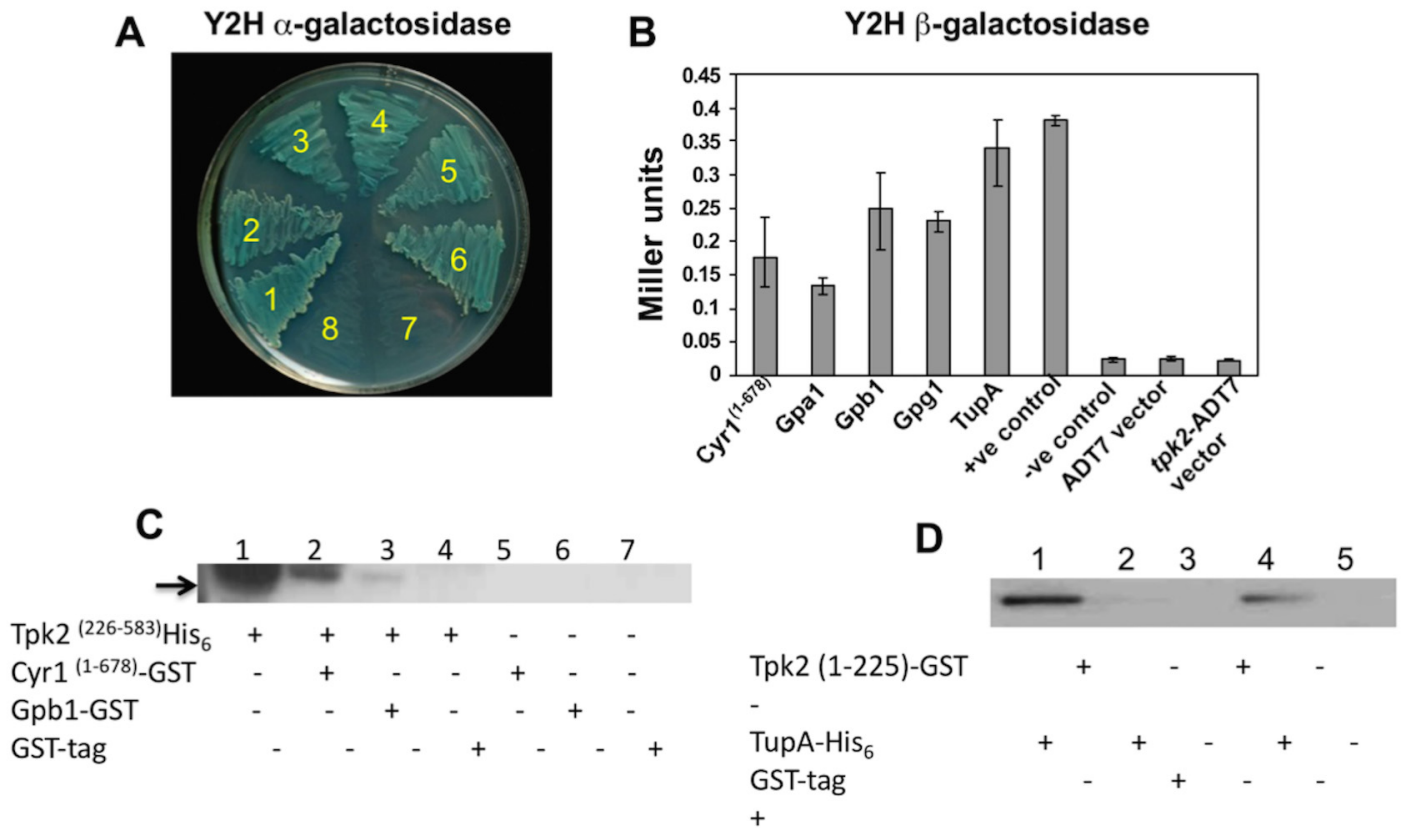
PbTpk2 interacts with the adenylate cyclase, G proteins and the co-regulator PbTupA. Yeast two-hybrid assays were performed to detect interactions between Tpk2 and other components of the cAMP-signaling pathway. The yeast strain AH109 was simultaneously transformed with the bait and prey vectors and plated out on SD/-Leu/-Trp for 3 days; yeast colonies that grew on SD/-Leu/-Trp were re-streaked on SD/-Ade/-His/-Leu/-Trp and incubated for a further 3 days to identify positive interactions. Positive colonies were confirmed by transfer to **(A)** SD/-Ade/-His/-Leu/-Trp plates supplemented with α-X-Gal and monitored for the development of blue coloured colony growth, establishing that PbTpk2 interacts with 1. Pb Cyr1^(1–678)^, 2. PbGpa1, 3. Pb Gpb1, 4. Gpg1, 5. TupA, 6. Positive control (pGBKT7-p53 + pGADT7-T), 7 and 8. Negative controls (pGBKT7-Lam + pGADT7-T and pGADT7-Tpk2); **(B)** SD/-Ade/-His/-Leu/-Trp broth for the growth of cells, which were subsequently collected and assayed for β-galactosidase activity with ONPG as substrate. The β-galactosidase activity of the cells transformed with the indicated yeast two-hybrid constructs is presented as a bar chart. These analyses indicated that PbCyr1^(1–678)^, PbGpa1, PbGpb1, PbGpg1 and PbTupA could interact with PbTpk2. In a positive control pGBKT7 p53 interacts with pGADT7-T and in a series of negative controls pGBKT7-*PbTPK2* could not interact with pGADT7-T, pGAD-*P53* ^(72–390)^ and pGAD-*Lam* ^(66–230^). **(C)** Pull-down assays to demonstrate that PbCyr1 and PbGpb1 both interact with PbTpk2. PbCyr1^(453–678)^–GST and PbGpb1-GST were overexpressed and purified from *E coli*, loaded on to glutathione sepharose beads before incubation with PbTpk2^(226–583)^-His_6_. After washing the beads, the proteins were eluted by the addition of NuPAGE 4X LDS sample buffer, followed by boiling at 90°C for 5 minutes, and separated on a 4–12% NuPAGE gel under denaturing conditions. Bound PbTpk2^(226–583)^-His_6_ was detected by Western-blotting using antibodies to the His-tag. Lane 1 PbTpk2^(226–583)^-His_6_ input protein, lanes 2 and 3 establish that PbTpk2 binds to immobilized fragments of PbCyr1 (lane 2) and PbGpb1 (lane 3), respectively. Negative controls in which PbTpk2^(226–583)^-His_6_, + GST-tag and PbCyr1^(453–678)^–GST and PbGpb1-GST, in the absence of PbTpk2^(226–583)^-His_6_, and GST-tag were added to glutathione sepharose beads are shown in lanes 4, 5, 6, and 7 respectively. **(D)** Pull-down assays to demonstrate that PbTupA interacts with PbTpk2. PbTpk2^(1–225)^ was purified from *E*. *coli*, loaded on to glutathione sepharose beads before incubation with PbTupA-His_6_. After washing the beads, eluted PbTupA-His_6_ was detected by Western-blotting (lane 1) using antibodies to the His-tag. A series of negative controls are shown in lanes 2,3 and 5. The input protein PbTupA-His_6_ is shown in lane 4.

Recent studies have shown that components of the cAMP-signaling pathway in *S*. *cerevisiae*, which are upstream of AC, can bypass AC and directly regulate the activity of PKA [38,39]. The kelch-repeat protein Krh1, a Gβ-mimic, was shown to interact with the catalytic subunits Tpk1-3, apparently enhancing their interaction with the regulatory subunit Bcy1 to reduce PKA activity [38]. Conversely, there is a hyper-accumulation of cAMP in *S*. *cerevisiae TPK*Δ mutants that suggests that PKA activity is required as part of a feedback mechanism to reduce cAMP levels [30,66–68]. Considering these features of ScTpk2, we sought to test if PbTpk2 interacted with any of the components of the cAMP-signaling pathway in *P*. *brasiliensis*. Using Y2H assays we detected interactions between PbTpk2 and the G proteins PbGpa1, PbGpb1 and PbGpg1 (Table 1), which we had previously found to interact with one another [19]. In contrast, we did not detect an interaction of PbTpk2 with PbGpa2 or PbGpa3 (Table 1), neither of which appear to interact with PbGpb1, PbGpg1 or PbAC [19]. We also found that PbTpk2, in common with PbGpa1, PbGpb1 and PbGpg1, interacted with the N-terminal domain of PbAC (e.g. PbCyr1^(1–678)^) (Fig 2A and 2B, Table 1) and PbGpa1, PbGpb1, PbGpg1 and PbCyr1^(1–678)^ all interacted with the C-terminus of PbTpk2 (e.g. PbTpk2^(265–583)^) (Table 1). Pulldown assays confirmed that PbTpk2^(226–583)^ interacted with both PbGpb1 and PbCyr1^(453–678)^) (Fig 2C). However, in contrast to PbGpb1, Y2H assays revealed that PbCyr1^(453–678)^) interacted with PbTpk2^(1–270)^, but not PbTpk2^(1–174)^. Although PbTpk1 also interacted with PbCyr1^(1–678)^ it did not interact with PbGpb1 (Table 1). These findings further support our previous proposal that the N-terminal domain of PbAC acts as a scaffold for the formation of a signaling complex [19].

**Table 1:** Yeast two-hybrid analyses–*P*. *brasiliensis*/*P*. *brasiliensis* gene interactions.

Interacting partners	PbTpk2^1-174^	PbTpk2^1-270^	PbTpk2^226-583^	PbTpk2^265-583^	PbTpk2^1-583^	PbTpk1	PbGpb1
**PbCyr1** ^**1-678**^	-	+	+	+	+	+	+
**PbGpa1**	-	-	NT	+	+	NT	+
**PbGpa2**	NT	-	NT	-	-	NT	-
**PbGpa3**	NT	-	NT	-	-	NT	-
**PbGpg1**	-	+	NT	+	+	NT	+
**PbGpb1**	-	-	+	+	+	-	NT
**PbTupA**	-	+	-	-	+	-	-

Colonies that grew on SD-Ade/-His/-Leu/-Trp drop-out plates and had α-galactosidase activity, as determined by a blue colouration of the colonies growing on α-X-gal supplemented media, are defined as positive (+) and those that did not as (-). All the gene sequences were swapped between the pGADT7 and pGBKT7 vectors and only those giving a positive-reaction in both vectors were scored as positive for an interaction. In addition, Tpk2^(1–583)^ was tested for interactions with PbCyr1^(600–1316)^, PbCyr1^(1302–1871)^, PbCyr1^(1649–2100)^ but none were detected. A set of control reactions was undertaken at the same time to validate the test reactions: positive control, pGADT7- Ag with pGBKT7-P53; negative controls, pGBKT7-Lam with pGADT7, pGADT7-TPK2^1-270^, pGADT7-TPK2^1-583^, pGADT7-TPK2^265-583^ and pGADT7-GPG1, and pGBKT7-TPK1 with pGADT7, pGBKT7, pGADT7-Lam, pGBKT7-Lam and pGBKT7-PbActin. NT- Not tested.

To test if these interactions were specific to the *P*. *brasiliensis* proteins we used Y2H assays to test for interactions between PbGpa1, PbGpb1 and PbTpk2^(1–583)^ and the N-terminal (Cyr1^(1–365)^), Gα-association (Cyr1^(350–480)^) and RAS-association (Cyr1^(600–800)^) domains of *S*. *cerevisiae* AC, to which PbGpa1, PbGpb1 and PbTpk2 bind in PbAC [19]. We could not detect any interaction of PbGpa1, PbGpb1 and PbTpk2 with these domains of ScAC (Table 2), suggesting that the *P*. *brasiliensis* proteins interact specifically with PbAC. In contrast, previous studies have revealed that SpGpa2 and ScGpa2 interact with the N-terminal domains of SpAC and ScAC from *Schizosaccharomyces pombe* and *S*. *cerevisiae*, respectively [38,69]. Similarly, we did not detect an interaction between PbGpb1 and ScTpk2 (Table 2). We also tested the GPCR ScGpr1^(679–961)^ for interactions with PbGpa1-3 and PbCyr1^(1–678)^; but it did not interact with any of the *P*. *brasiliensis* proteins (Table 2). In contrast, we were able to confirm that ScGpr1^(679–961)^ interacts with ScGpa2, which interacts with ScCyr1^(350–480)^ (Table 2). The lack of interaction of the *P*. *brasiliensis* proteins with the *S*. *cerevisiae* proteins suggests that these fungi require a specific set of components for detection and propagation of the signal to AC/PKA.

**Table 2:** Yeast two-hybrid analyses—*P*. *brasiliensis*/*S*. *cerevisiae* gene interactions.

Interacting partners	PbGpa1	PbGpb1	PbTpk2	PbTupA	ScGpa2	ScBcy1
**ScCyr1** ^**1-365**^	-	-	-	NT	-	NT
**ScCyr1** ^**350-480**^	-	-	-	NT	+	NT
**ScCyr1** ^**600-800**^	-	-	-	NT	-	NT
**ScTpk2**	-	-	-	-	NT	+
**ScBcy1**	NT	NT	+	NT	NT	NT
**ScGpr1** ^**679-961**^	-	-	NT	NT	+	NT

Colonies that grew on SD-Ade/-His/-Leu/-Trp drop-out plates and had α-galactosidase activity, as determined by a blue colouration of the colonies growing on α-X-gal supplemented media, are defined as positive (+) and those that did not as (-). All the gene sequences were swapped between the pGADT7 and pGBKT7 vectors and only those giving a positive-reaction in both vectors were scored as positive for an interaction. The ScCyr1^(1–365)^, ScCyr1^(350–480)^, ScCyr1^(600–800)^ are the N-terminal, Gα and Ras association domains, respectively; whilst ScGpr1(679–961) the ScGpa2 association domain. A set of control reactions was undertaken at the same time to validate the test reactions: positive control, pGADT7- Ag with pGBKT7-P53; negative controls, pGBKT7-Lam with pGADT7, pGADT7-TPK2^1-270^, pGADT7-TPK2^1-583^, pGADT7-TPK2^265-583^ and pGADT7-GPG1, and pGBKT7-TPK1 with pGADT7, pGBKT7, pGADT7-Lam, pGBKT7-Lam and pGBKT7-PbActin. NT- Not tested.

### PbGpb1 inhibits the function of PbTpk2 in stimulating filamentation

**Fig 3:**
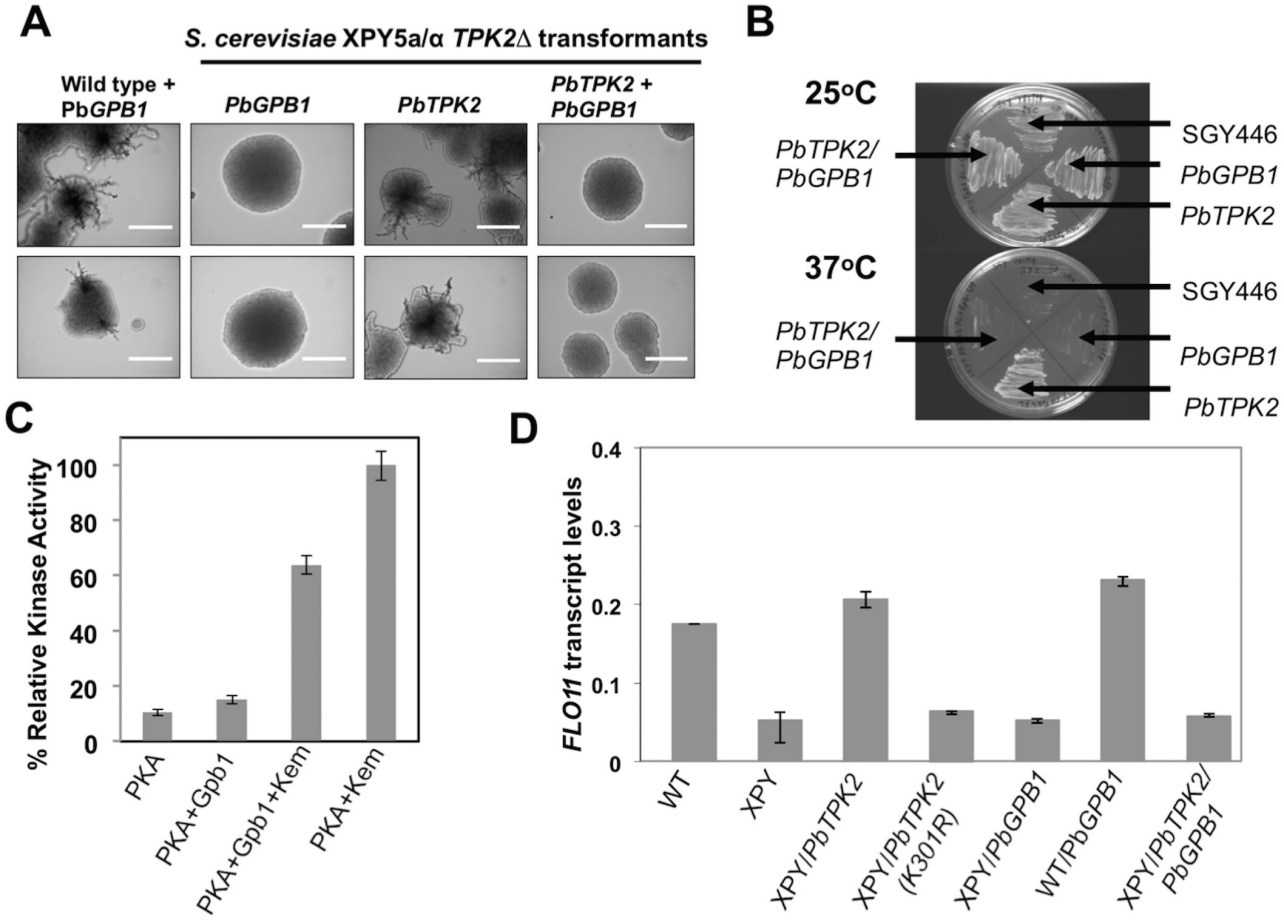
The Gβ protein Gpb1 blocks Tpk2-induced pseudohyphal differentiation. **(A)** The *S*. *cerevisiae* diploid strain XPY5a/α, which had been transformed with *PbTPK2* ^*(1–583)*^, was transformed with a construct for the expression of PbGpb1-GFP and the transformants, selected on the basis of their green fluorescence, analysed for pseudohyphal growth in SLAD agar containing 50 μM (upper panel) or 200 μM (lower panel) ammonium sulfate. Single colonies from the agar plate were observed at 20x magnification in an Eclipse E-400 microscope. The scale bar is 50μm. Whilst control cells expressing PbTpk2 were able to form pseudohyphae, those co-transformed with *PbGPB1* could not. As a control the wild-type strain MLY61a/α was transformed with *PbGPB1* and shown to still produce pseudohyphae. **(B)** The *S*. *cerevisiae* haploid strain SGY446, which had been transformed with *PbTPK2* ^*(1–583)*^, so that it could grow at 37°C, was transformed with a construct for the expression of PbGpb1-GFP and the transformants, selected on the basis of their green fluorescence after growth at 25°C. The SGY446/*PbTPK2* ^*(1–583)*^/*PbGPB1-GFP* transformant was tested for growth at 37°C. In contrast to the control strain SGY446/*PbTPK2* ^*(1–583)*^, the SGY446/*PbTPK2* ^*(1–583)*^/*PbGPB1-GFP* transformant could not grow at 37°C. **(C)** A bar chart showing the relative *in vitro* kinase activity of *P*. *brasiliensis* Tpk2 (PKA) using kempeptide and PbGpb1 as substrates. Note that a slight excess of PbGpb1 causes about a 40% reduction in the kinase activity of PbTpk2 using kempeptide as substrate. **(D)** A bar chart showing *FLO11* transcript levels for the indicated transformants. The measured quantity of the *FLO11* mRNA in each of the treated samples is the relative abundance to the value for *actin*. The data represent the average of 3 measurements.

In order to determine the effect of PbGpb1 on PbTpk2 we sought to exploit the fact that the *S*. *cerevisiae* cAMP-signaling pathway does not utilize true Gβ proteins and would, presumably, be unresponsive to them. In accord with this assumption, the expression of *PbGPB1* in WT MLY61a/α and *tpk2Δ*- XPY5a/α *S*. *cerevisiae* had no apparent effect on the morphology of the cells (Fig 3A). Furthermore, PbGpb1 did not bind to ScAC or ScTpk2 (Table 2), and consistent with the lack of interaction, the overexpression of *PbGPB1* in MLY61a/α and XPY5a/α *S*. *cerevisiae* did no cause any change in *FLO11* transcript levels (Fig 3D), as would be expected if the cAMP-signaling pathway was either stimulated or inhibited. In contrast, when the *S*. *cerevisiae* XPY5a/α and SGY446 strains, which had been transformed with *PbTPK2* ^*(1–583)*^ (or *PbTPK2* ^*(226–583)*^ (data not shown)), were used as recipients for transformation with a *PbGPB1-GFP* fusion construct; we found that the co-expression of PbTpk2 with PbGpb1 inhibited the ability of the XPY5a/α co-transformants to produce pseudohyphae (Fig 3A), whilst the SGY446 co-transformants were unable to grow at 37°C (Fig 3B). Our data is consistent with PbGpb1 interacting with the C-terminal, catalytic, domain of PbTpk2 to directly inhibit its activity. To test this hypothesis we measured the transcript levels for *FLO11* for XPY5a/α cells transformed with *PbTPK2*, and with *PbTPK2*/*PbGPB1*, under conditions of nitrogen-starvation to stimulate production of pseudohyphae; consistent with our hypothesis, the *PbTPK2* transformants had significantly higher *FLO11* levels than the *PbTPK2/PbGPB1* transformants (Fig 3D).

**Fig 4:**
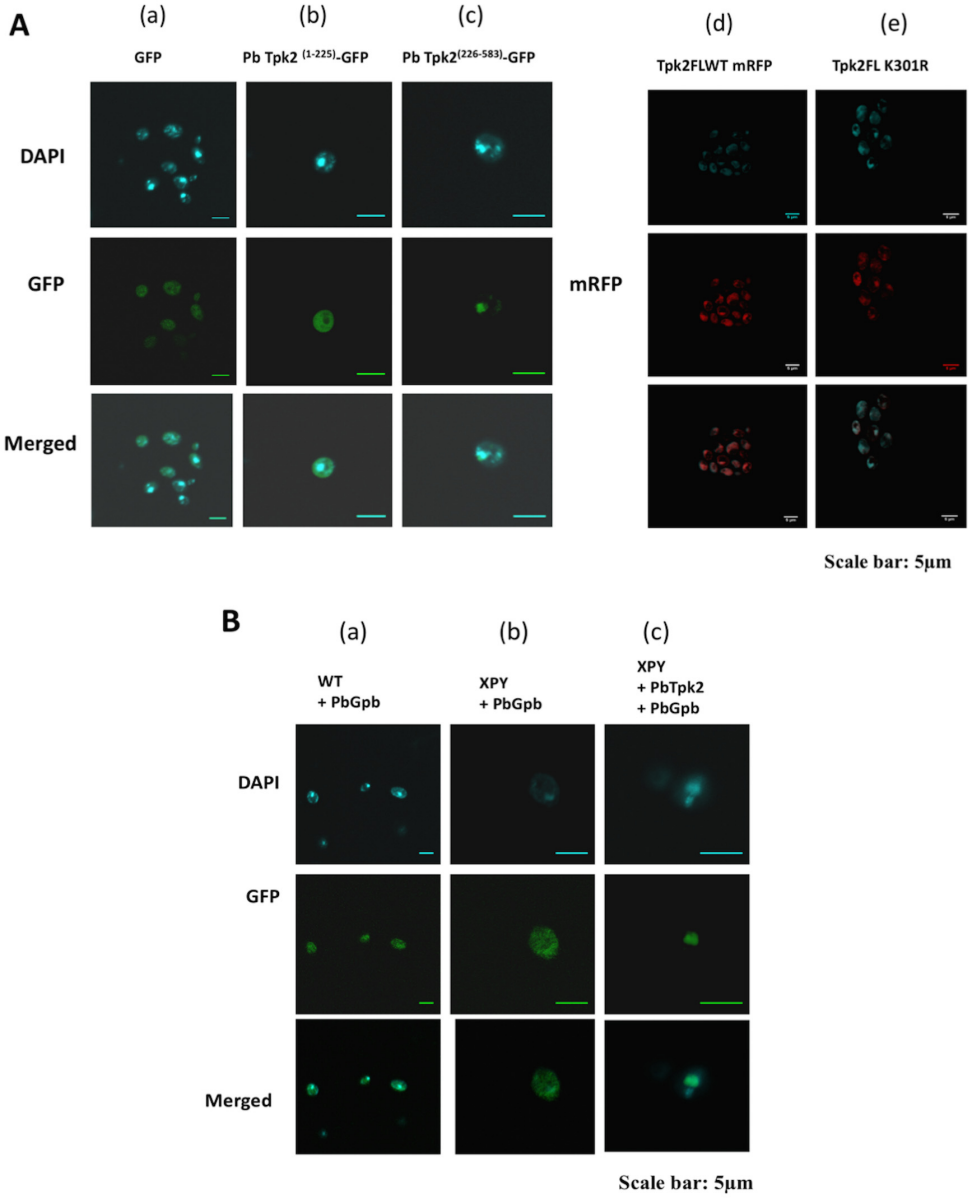
The subcellular localization of PbTpk2 and PbGpb1: PbGpb1 is targeted to the nucleus in cells expressing PbTpk2. **(A)** Cells of *S*. *cerevisiae* XPY5a/α (*TPK2Δ* mutants) were transformed with constructs for the expression of either PbTpk2-GFP or PbTpk2-mRFP fusion proteins and the localization of the proteins detected by confocal microscopy using GFP or mRFP as a marker. The nuclei of the yeast cells were identified by staining with DAPI for confocal microscopy. (a) GFP-expressed from p426MET25, and PbTpk2 N-terminus 1-225-GFP (b) were distributed throughout the cell; PbTpk2 C-terminus 226-583-GFP (c), PbTpk2 FL-mRFP (d) and PbTpk2 (K301R) FL-mRFP (e) were concentrated in the nucleus. **(B)** Cells of the *S*. *cerevisiae* XPY5a/α/ *PbTPK2* transformant were transformed with a construct for the expression of the Gpb1-GFP fusion protein and the localization of the protein detected by confocal microscopy using GFP as a marker. (a) Gpb1-GFP was distributed throughout the cell in the wild-type (MLY61a/α) strain; (b) Gpb1-GFP was distributed throughout the cell in the XPY5a/α (*ΔTPK2* mutant) strain; and (c) Gpb1-GFP was concentrated in the nucleus when co-expressed with (full-length) *P*.*brasiliensis* Tpk2 (PbTpk2FL) in the *TPK*2Δ *S*. *cerevisiae* cells. In (A) and (B) the upper panel is for the nuclei stained with DAPI, the middle panel for GFP (or mRFP) and the lower panel is the merged DAPI and GFP (or mRFP) images. The scale bar is 5 μm. (C) The localization of PbGpb1, expressed in XPY5a/α (*TPK2Δ*) *S*.*cerevisiae* cells, was determined by immune-gold electron microscopy using antibodies to PbGpb1, revealing that PbGpb1 is localized at the cell membrane, and in the cytoplasm and nucleus.

We considered the possibility that the PbGpb1 inhibitory function is due to its ability to re-target PbTpk2 from the nucleus to the cytoplasm, preventing phosphorylation of transcription factors involved in pseudohyphal development. Using GFP- and/or mRFP-fusion proteins for protein localization in cells, we established that PbTpk2^(1–583)^ and PbTpk2^(226–583)^ are targeted to the nucleus, whilst PbTpk2^(1–225)^ (Fig 4A) and PbGpb1 are distributed through the cell (Fig 4B). However, in cells co-transformed with both *PbTPK2* ^*(1–583)*^, or *PbTPK2* ^*(226–583)*^, and *PbGPB1*, PbGpb1 was also targeted to the nucleus (Fig 4B). Immuno-gold electron-microscopy confirmed that PbGpb1 is targeted to the nucleus of *S*. *cerevisiae* in the presence of PbTpk2 (data not shown). Our confocal studies suggest that there is a redistribution of PbGpb1 to the nucleus with PbTpk2. This behavior would be consistent with PbGpb1 inhibiting PbTpk2 from phosphorylating transcription factors in the nucleus that are necessary for the production of pseudohyphae. We sought to test if PbGpb1 was a substrate for PbTpk2 and whether its phosphorylation was required for its nuclear targeting and inhibitory effect. PbGpb1 is predict to have two potential PKA phosphorylation sites (e.g. S109 and S151); mutation of these sites (e.g. to arginines) to block phosphorylation did not prevent the targeting of PbGpb1 to the nucleus, nor its ability to repress the production of pseudohyphae by the XPY5a/α/*PbTPK2* transformants (data not shown). This data would argue against PbGpb1 simply acting as a substrate of PbTpk2 that competitively inhibits the phosphorylation of substrate transcription factors; although we cannot exclude the possibility that it binds non-productively to the active site of PbTpk2. Similarly, we tested and found that PbTpk2 (K301R) is tageted to the nucleus, indicating that the decrease in Tpk2 activity is not merely due to mislocalization of the protein in the cytoplasm (Fig 4). We also found that PbGpb1 coexpressed with PbTpk2 (K301R) was still targeted to the nucleus, further indicating that its phosphorylation by PKA was not required to effect its relocalization (data not shown).

In order to determine if PbGpb1 had any effect of on the activity of PbTpk2 these proteins were overexpressed and purified from *E*. *coli* for use in kinase assays. PbTpk2 was found to have kinase activity using kemptide as a substrate for phosphorylation (Fig 3C), which could be abolished by the addition of a PKA inhibitory peptide (Upstate) (data not shown). Using the same assay, we found that PbGpb1 was a poor substrate of PbTpk2; however, when (3 μM) PbTpk2 was incubated with the (50 μM) kemptide and (4 μM) PbGpb1 there was a 40% reduction in kinase activity (Fig 3C), indicating that PbGpb1 acts as an inhibitor of PbTpk2. Considering that the PbGpb1 was at a much lower concentration than the kemptide would argue against it merely competing with the kemptide for the active site of PKA.

In contrast to PbGpb1, we found that although PbGpa1 interacted with PbTpk2, it did not co-localize with PbTpk2 to the nucleus and was unable to block the pseudohyphal growth of the *S*. *cerevisiae* XPY5a/α/*PbTPK2* transformants (S4 Fig), establishing that PbGpb1 acts specifically to switch the cells between filamentous and yeast growth and consistent with our proposal that nuclear targeting of PbGpb1 may be necessary for its inhibitory effect on PbTpk2. Furthermore, in contrast to PbTpk2, PbTpk1 was not targeted to the nucleus but was localized within distinct structures within the cytoplasm (S3 Fig), which is in line with recent studies showing that *S*. *cerevisiae* Tpk proteins can be targeted to P-bodies in the cytoplasm [70].

### PbTpk2 interacts with PbTupA

In a screen of a *P*. *brasiliensis* cDNA two-hybrid library in yeast, using *PbTPK2* ^*(1–270)*^ as the bait protein, we isolated a cDNA with homology to *TUP* genes. Subsequently we isolated the cDNA for this gene from our pDNR library and completed its sequencing by walking on both the cDNA and genomic DNA. This revealed a protein, which was predicted to have an N-terminal coiled-coil and seven WD40 repeat sequences, with a high-degree of sequence similarity to Tup in other fungi (S5 Fig). For example, it has 79% identity to TupA from *Penicillium marneffei*, which has been shown to promote filamentous growth [56]. We used Y2H assays to confirm the interaction of *P*. *brasiliensis* TupA with PbTpk2 and established that it binds to a region between residues 174–226 of PbTpk2 (Fig 2A and 2B; Table 1). As further confirmation of the interaction, a pulldown assay was used to demonstrate an interaction between PbTupA and PbTpk2^(1–225)^–GST (Fig 2D). In contrast, PbTpk1 did not interact with PbTupA (Table 1).

### PbTupA induces filamentous growth of *S*. *cerevisiae*

**Fig 5:**
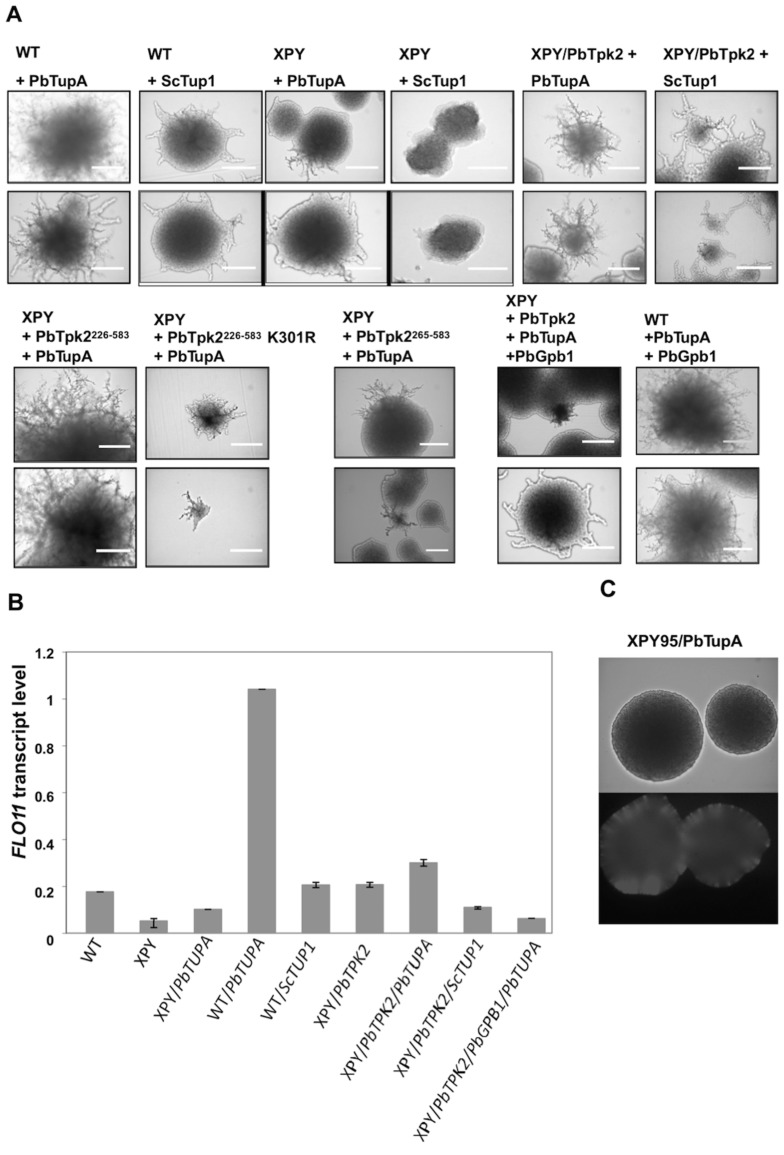
PbTupA induces hyperfilamentous growth that can be repressed by PbGpb1. The *S*. *cerevisiae* diploid strain MLY61a/α (WT) and its *TPK2Δ* mutant XPY5a/α (XPY) were transformed with the *PbTUPA*, *ScTUP1*, *PBTPK2* and *PbGPB1* as indicated. To allow selection, generally, constructs for the expression of the PbTupA-mRFP, ScTup1-mRFP, PbTpk2-GFP and PbGpb1-GFP fusion proteins were used and the transformants, selected on the basis of their green and/or red fluorescence. **(A)** The cells were analysed for pseudohyphal growth in SLAD agar containing 50 μM (upper panel) or 200 μM (lower panel) ammonium sulfate. Single colonies from the agar plate were observed at 20x magnification in an Eclipse E-400 microscope. The scale bar is 50μm. WT cells expressing PbTupA were hyperfilamentous; whilst those expressing ScTup1 did not produce pseudohyphae. XPY cells expressing PbTupA produced few pseudohyphae; whilst those expressing PbTupA with PbTpk2, but not a kinase defective K301R derivative, were hyperfilamentous, indicating the requirement for a functional PKA. The co-expression of PbGpb1 with PbTupA repressed the filamentous growth of the XPY/*PBTPK2* but not the WT cells, indicating that PbGpb1 specifically inhibits PbTpk2. **(B)** A bar chart showing *FLO11* transcript levels for the indicated transformants. The measured quantity of the *FLO11* mRNA in each of the treated samples is the relative abundance to the value for *actin*. The data represent the average of 3 measurements. **(C)** The *S*. *cerevisiae* diploid strain XPY95a/α, a *FLO8Δ* mutant, was transformed with the *PbTUPA-mRFP and the transformants* selected on the basis of their red fluorescence (lower panel), and analysed for pseudohyphal growth in SLAD agar containing 50 μM (upper panel) ammonium sulfate. PbTupA was in capable of inducing either the development of pseudohyphae (upper panel) or invasive growth (lower panel), which are the hallmarks of the parent strain MLY61a/α (WT) transformed with *PbTUPA-mRFP*. This data suggests that PbTupA works up-stream of Flo8 to induce hyperfilamentous growth.

Whilst the expression of *PbTUPA* in *S*. *cerevisiae* MLY61a/α caused the cells to become hyperfilamentous, few pseudohyphae were produced by XPY5a/α cells transformed with *PbTUPA* (Fig 5A and S6 Fig). Similarly, we found that XPY5a/α cells co-transformed with *PbTPK2* and *PbTUPA* were hyperfilamentous, but those co-transformed with a kinase defective K301R derivative of *PbTPK2* and *PbTUPA* produced few pseudohyphae (Fig 5A and S6 Fig). The degree of filamentation correlated with the *FLO11* transcript levels; for the MLY61a/α/*PbTUPA* cells these were about 3-fold higher than for the XPY5a/α/*PbTPK2*/*PbTUPA* cells (Fig 5B), which were about 2-fold higher than for the untransformed MLY61a/α cells. These data indicate that PbTupA functions primarily, but not exclusively, through the cAMP-signaling pathway, down-stream of functional Tpk2, to induce *FLO11* expression and pseudohyphae formation. Indeed, PbTupA was unable to induce the hyperfilamentous growth of a *FLO11Δ S*. *cerevisiae* strain XPY107a/α [20] (data not shown). We found that in common with Tpk2, PbTupA is targeted to the nucleus (S7 Fig), where presumably they form a complex with the relevant transcription factors to induce Flo11 expression that leads to pseudohyphal production.

Considering that PbTupA was isolated by its interaction with the N-terminal domain of PbTpk2, apparently due to its binding between residues 174–226 of PbTpk2; we decided to test if the N-terminal truncated PbTpk2 was also functional with PbTupA in promoting hyperfilamentous growth and agar invasiveness. The cells expressing *PbTUPA* with *PbTPK2* ^*(226–583)*^, which lacks the first glutamine-rich region, were hyperfilamentous; but those expressing *PbTUPA* with *PbTPK2* ^*(265–583)*^, which lacks both glutamine-rich domains, produced some pseudohyphae but neither hyperfilamentous nor agar invasive growth (Fig 5A and S6 Fig). This data indicates that PbTupA needs to interact with the N-terminus of PbTpk2 to be functional. Interestingly, PbTupA can function with both PbTpk2 and ScTpk2, which lacks an extensive N-terminal domain (S2 Fig). Although much shorter, it is notable that the N-terminal domain of ScTpk2 includes a glutamine-rich domain (S2 Fig), but we did not detect an interaction between ScTpk2 and PbTupA (Table 1). (Possibly TupA’s hyperfilamentation phenotype requires either active Pb Tpk2 or Sc Tpk2 in addition to binding to the N-terminus). There is the possibility that a *S*. *cerevisiae* protein, such as Ssn6, bridges PbTupA and ScTpk2.

These results were surprising because in *S*. *cerevisiae* Tup1 represses gene expression [71] and work specifically with Sfl1 to block filamentous growth [44]. Consequently, we tested whether expressing ScTup1, from the same p426MET25 vector used to express PbTupA, would induce hyperfilamentous growth of *S*. *cerevisiae* MLY61a/α and/or XPY5a/α cells. The MLY61a/α/*ScTUP1* cells produced only a few pseudohyphae (repressed) and XPY5a/α/*ScTUP1* cells produced no pseudohyphae, indicating that the ability to induce hyperfilamentous growth is a specific feature of PbTupA. Broadly consistent with this behaviour, and some studies that revealed that *ScTUP1*-deletion strains can have lower *FLO11* expression levels [72], the *FLO11* transcript levels for the MLY61a/α/*ScTUP1* cells were at a similar level to those for untransformed MLY61a/α cells (Fig 5B). Interestingly, XPY5a/α/*PbTPK2*/*ScTUP1* cells had much lower *FLO11* transcript levels, compared to XPY5a/α/*PbTPK2* cells, and produced very few, if any, pseudohyphae (Fig 5A and 5B), suggesting that ScTup1 can supress pseudohyphae formation via its interaction with Tpk2. This behaviour is consistent with early studies that indicated that ScTup1 overexpression alone caused gene repression [71]. Indeed, there is about a 3-fold and a 5-fold difference in the *FLO11* transcripts levels for XPY5a/α/*PbTPK2/PbTUPA* and XPY5a/α/*PbTPK2/ScTUP1* and for MLY61a/α/*PbTPK2/PbTUPA* and MLY61a/α/*PbTPK2/ScTUP1*, respectively, which would suggest that these co-repressors have opposing effects (Fig 5B).

One interesting possibility is that, in contrast to ScTup1, which works with Sfl1, PbTupA works with Flo8, to stimulate *flo11* transcription, causing hyperfilamentous growth. To test this possibility, we expressed PbTupA in the *FLO8Δ* strain XPY95a/α [20]; revealing that PbTupA was unable to induce hyperfilamentous growth (Fig 5C). Our data indicates that PbTupA functions down stream of Tpk2 but up-stream of Flo8.

### PbGpb1 over-rides the effect of PbTupA to repress filamentous growth

Considering that PbGpb1 was capable of blocking the production of pseudohyphae by XPY5a/α cells transformed with *PbTPK2*; we sought to test if it could block the hyperfilamentous growth of XPY5a/α/*PbTPK2* cells transformed with *PbTUPA*. Cells expressing *PbTPK2*, *PbTUPA* and *PbGPB1* produced few pseudohyphae that were seen only rarely (Fig 5A and S6 Fig); and these cells had lower *FLO11* expression levels than cells expressing *PbTPK2* and *PbTUPA* (Fig 5B). This inhibition cannot be attributed to a direct effect of PbGpb1 on PbTupA because no interaction of these proteins was detected (Table 1), suggesting that PbGpb1 curtails *FLO11* transcript levels to block hyperfilamentation via its interaction with PbTpk2. PbGpb did not Interact with ScTpk2 (Table 2), presumably the reason why Gpb1 has no effect on WT MLY61a/α (Fig 3A). This proposal was further substantiated by the finding that PbGpb1 did not block the hyperfilamentous growth of MLY61a/α cells transformed with *PbTUPA* and consistent with it acting specifically through PbTpk2 (Fig 5A and S6 Fig).

### PbTupA induces invasive growth and production of an aerial stalk

**Fig 6:**
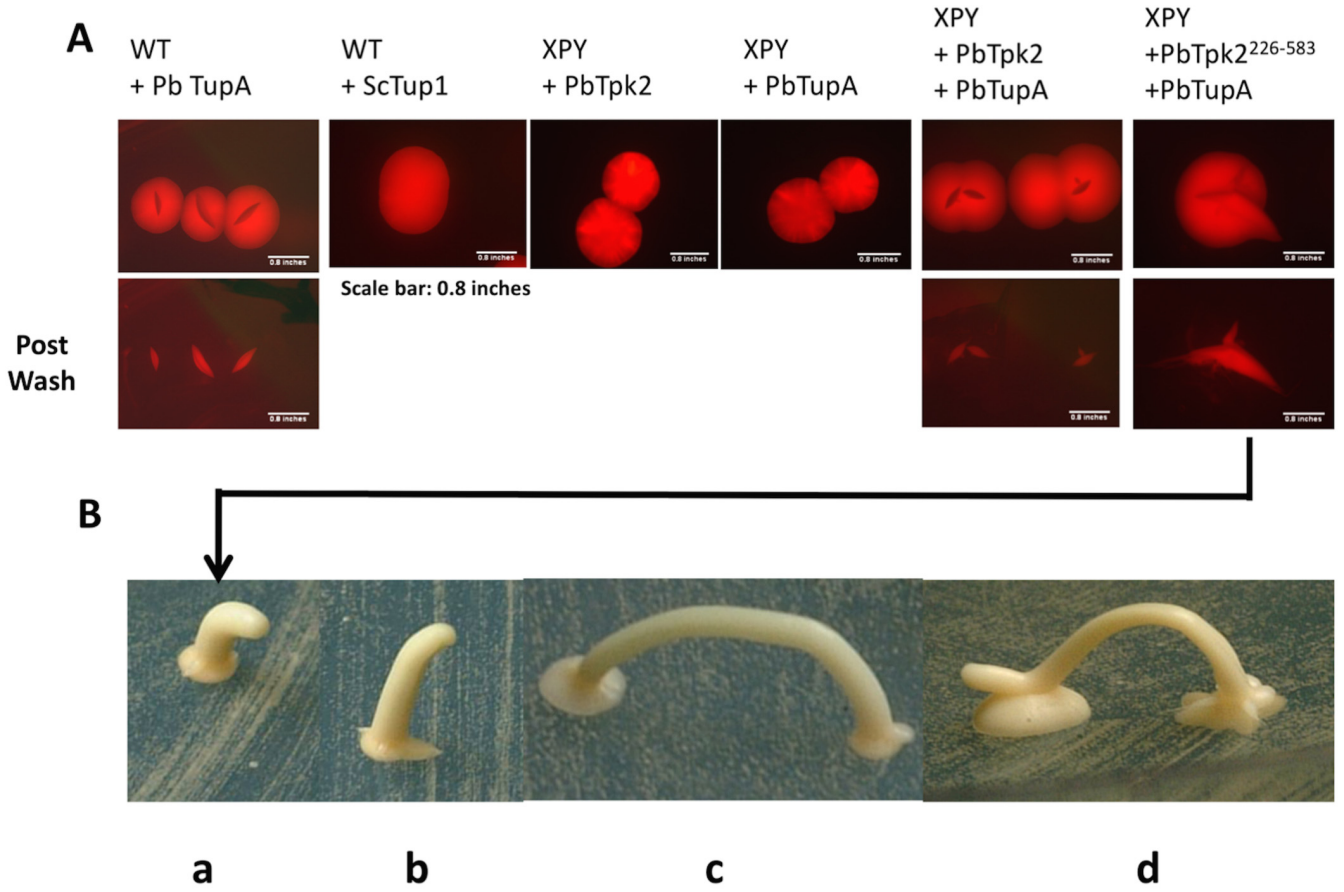
The expression of *PbTUPA* causes invasive and aerial growth of *S*.*cerevisiae*. **(A)** The *S*. *cerevisiae* diploid strains MLY61a/α (WT) and XPY5a/α, a *TPK2Δ* mutant, were transformed with the *PbTUPA*, *ScTUP1* and *PBTPK2* as indicated. To allow selection, generally, constructs for the expression of the PbTupA-mRFP, ScTup1-mRFP and PbTpk2-mRFP fusion proteins were used and the transformants, selected on the basis of their fluorescence. The colonies were viewed by Leica M165 FC stereo fluorescence microscopy to identify the invasive growth on the plates. The colony views are shown both before (top panel) and after (bottom panel) washing to remove cells that had not invaded the agar. In response to the expression of PbTupA, WT cells and XPY cells transformed with *PbTPK2* produced invasive growth. XPY cells expressing PbTupA did not invade the agar, indicating that functional PbTpk2 was required for this phenotype. WT cells expressing ScTup1were non-invasive. **(B)** XPY cells expressing PbTpk2 and PbTupA developed an aerial stalk (a-c) that topples over to start a new colony (c) and to develop a second stalk (d).

Interestingly, when *PbTUPA* was expressed in MLY61a/α or XPY5a/α/*PbTPK2* diploid cells two other phenotypes became apparent upon prolonged growth of a week or more: firstly the cells clearly became invasive, with a thick shaft, emanating from the colony centre, entering the agar and radiating outwards (Fig 6A and S6 Fig). Previous studies have reported that *S*. *cerevisiae* diploid cells can become invasive in response to overexpression of Flo11 [42]. Therefore this behaviour is consistent with the high levels of *FLO11* transcripts in these cells (Fig 5B). The colony, having developed an invasive shaft, then went on to develop an aerial projection, visible by eye, which grew for several millimetres before it apparently became top-heavy and toppled over, to initiate a new colony upon contact with the agar (Fig 6B). Previous studies have reported that very rarely *S*. *cerevisiae* colonies can be induced to form stalks that resemble the aerial projections we have identified [73,74]. Our findings suggest these stalks may be for colony transfer since the stalk can be used to imprint a copy of the colony at a distant position (Fig 6B(d)). Importantly, the new colony can develop quickly because a number of cells are simultaneously transferred. This behaviour was dependent upon PbTupA because cells expressing PbTpk2^(226–583)^ were invasive and produced a stalk; whilst those expressing PbTpk2^(265–583)^ were non-invasive and did not develop a stalk (Fig 6 and S6 Fig). Furthermore, cells expressing ScTup1 were also non-invasive (S6 Fig).

In an attempt to determine the role of the *PbGPB1* and *PbTUPA* genes we made antisense-RNA constructs but we were unable to knock-down these genes in *P*. *brasiliensis* (data not shown).

## Discussion

Previously we established that the cAMP-signaling pathway is important in controlling the morphological transition from mycelium to the pathogenic yeast form of *P*. *brasiliensis* [19]. Importantly, these studies indicated that there is an imbalance in the G-protein subunits during the mycelium to yeast transition, with the Gβ-protein PbGpb1 predominating. PbGpb1 can bind to adenylate cyclase PbAC and the imbalance in its expression correlated with a decrease in cellular cAMP. We proposed that activation of the cAMP-signaling pathway in mycelium would rely on GTP-induced dissociation of the Gαβγ trimer to release the Gα-protein PbGpa1 that would activate PbAC; subsequently, there would be a shift to PbGpb1 signaling, which could bind PbAC to attenuate cAMP production. In *S*. *cerevisiae*, the Gβ-mimic Asc1 has been shown to function in a similar manner, binding to AC to inhibit cAMP production and attenuating cAMP-signaling [57]. For *P*. *brasiliensis*, this would provide a strategy to avoid the metabolic waste of using GTP to maintain the signal during the morphological transition, which can take 10 days for completion. However, our current studies suggest that PbGpb1 has a more fundamental role in controlling the morphological transition by interacting with the catalytic subunit of protein kinase A, PbTpk2 (Figs 2 and 3). In *S*. *cerevisiae*, the Gβ-mimic Krh1 has been shown to attenuate the activity of Tpk2 by causing it to reassociate with the Bcy1 regulatory subunit [38,39]. Our data would argue against PbGpb1 working in an analogous manner to Krh1, because PbTpk2 was overproduced and presumably would have been at higher levels than Bcy1 in *S*. *cerevisiae*. However, we found that PbTpk2 could interact with ScBcy1 and so we cannot fully exclude the possibility that PbGpb1 induces PbTpk2 to interact with ScBcy1, potentially preventing it from binding PbTupA. Irrespective of the mechanism of inhibition, the activity of PbTpk2, as determined from *FLO11* transcript levels, is clearly curtailed by PbGpb1 (Fig 3). This is the first demonstration that a fungal Gβ-protein can regulate the activity of PKA to block morphological changes (Fig 3).

G-proteins are generally thought to signal at the plasma-membrane, but there is increasing evidence that they are also present at intracellular compartments. Our confocal microscopy studies indicated that PbGpb1 is distributed throughout the cell but is concentrated in the nucleus in the presence of PbTpk2 (Fig 4B). Although fungal PKAs are known to be involved in phosphorylating a number of proteins to affect their targeting at the nucleus [75–77]; the accumulation of PbGpb1 at the nucleus in the presence of PbTpk2 did not appear to be dependent upon its phosphorylation by PbTpk2, but is instead most likely due to it being tethered there by PbTpk2. The localization of PbGpb1 may be an important effector of its function and this may well be determined by its interaction with other proteins such as PbTpk2, PbAC and PbGpg1, which localize predominantly to the nucleus [70], cytoplasm [30] and plasmamembrane [78], respectively. Recent studies have shown that Gα proteins are segregated into distinct pools that allow specific signaling pathways to be activated at the plasmamembrane and at intracellular membranes; for example, ScGpa1 that couples to pheromone receptors at the plasmamembrane can also interact with the phosphatidylinositol 3-kinase Vps34 and the Gβ-mimic VPS15, found at endosomal membranes [79,80]. Clearly, the positioning of PbGpb1 would be consistent with its interactions with Gpa1/Gpg1 at the membrane, with AC in the cytoplasm and Tpk2 in the nucleus, but the localization to specific foci might underlie additional, as yet unidentified, roles. Indeed, the Gβ-mimic Asc1 has a dual role to bind and regulate AC and ribosomes [57,58,81–85].

In multicellular organisms, A kinase anchoring proteins (AKAPs) target PKA holoenzymes to specific subcellular locations, and are thought to confer spatio-temporal control of PKA signaling in order to phosphorylate specific localized substrates [86–88]. Recently, AKAP proteins have been shown to act as scaffolds for signalosomes composed of AC and up- and down-stream components of the signaling pathway [89]. However, AKAP proteins have not been identified in fungi and, consequently, our knowledge of how spatio-temporal control of cAMP-signaling is achieved in fungi is limited. Interestingly, both fungal AC and PKA proteins have large N-terminal domains that are not present in the mammalian proteins. We hypothesised that these domains could acts as a scaffold for signalosome complexes in analogy to the role of AKAP proteins in mammals. Our studies revealed that PbGpa1, PbGpb1 and PbTpk2 all bind to the N-terminal domain of PbAC (Fig 2 and Table 1). In *S*. *cerevisiae*, Gpa2 [38], Acs1 [57] and Ras [90,91] have all been shown to bind to the N-terminal domain of ScAC; whilst the CAP [92] protein, which binds actin, binds to the C-terminal domain. In screening for proteins that interact with the N-terminus of PbTpk2, we discovered an interaction with the TupA co-repressor (Fig 2 and Table 1). Whilst TupA has a similar structure to Gpb1, both being WD or β-propeller proteins [93], our Y2H studies mapped them to different binding-sites on Tpk2, with TupA and Gpb1 binding to the N- and C-terminal domains respectively (Table 1). Considering that both Tpk2 and Tup proteins interact with a number of transcription factors suggests that Tpk2 can act as a scaffold for assembly of a transcriptional complex. Indeed, recent studies that have revealed that the terminal kinase in a pathway is physically associated with the chromatin at the position of the genes that are regulated [94]. Herein we provide the first evidence that the N-terminal domain of a fungal PKA can form a complex with the down-stream transcriptional machinery.

In contrast to PbTupA, *S*. *cerevisiae* cells over-expressing *ScTUP1* had lower *FLO11* transcript levels and little capacity for filamentous growth (Fig 5 and S6 Fig), in accord with previous studies that indicated that ScTup1 suppresses pseudohyphal growth [44]. In *S*. *cerevisiae*, Tup1 binds Ssn6, to form a co-repressor complex that represses multiple subsets of genes when recruited to promoters by sequence-specific DNA binding repressors [96]. The Tup1-Ssn6 co-repressor complex has been shown to interact with the transcriptional repressor Sfl1, which represses *FLO11* expression [43] and pseudohyphal growth [44], apparently via an interaction with components of the RNA polymerase sub-complex [95–96]. However, Sfl1 is also a target for Tpk2 and its phosphorylation causes derepression of *FLO11* transcription [43,44]. It is plausible that PbTupA triggers the hyperfilamentous growth of *S*. *cerevisiae* by specifically recruiting Sfl1 to Tpk2, promoting its phosphorylation. On the other hand, there is evidence that the Tup1-Ssn6 complex can work with some transcriptional activators to activate rather than repress gene expression [97]. The transcriptional activator Flo8 is a target of Tpk2 and its phosphorylation activates *FLO11* transcription [44]. An alternative and interesting possibility is that the Tpk2/PbTupA complex recruits Flo8, to activate *FLO11* transcription and hyperfilamentous growth. Consider that *S*. *cerevisiae* cells expressing PbTupA had about a 5-fold enhancement in *FLO11* transcript levels would suggest that the expression of the *FLO11* gene is being activated (Fig 5B), especially when considered along side previous studies that indicated that *FLO11* levels are at best doubled in an *SFL1* strain [44]. Moreover, deletion of Sfl1 has not been reported to confer the dramatic phenotypes, such as the invasive peg and aerial stalk, which occurs upon overexpression of PbTupA in *S*. *cerevisiae*. Consistent with the hypothesis that PbTupA recruits Flo8, we found that PbTupA was unable to induce the pseudohyphal growth of a *FLO8Δ* strain under nitrogen-starvation conditions (Fig 5C).

Considering that PbGpb1 interacted specifically with PbTpk2 to block pseudohyphal growth, we tested whether it could override the effect of PbTupA. *S*. *cerevisiae* cells transformed with *PbTPK2*, *PbTUPA* and *PbGPB1* had reduced levels of *FLO11* and did not form pseudohyphae (Fig 5 and S6 Fig). We did not detect a direct interaction of PbTupA and PbGpb1 and since PbTupA and PbGpb1 bind to the N- and C-terminal domains of PbTpk2 (Table 1), respectively; this data suggests that PbTupA and PbGpb1 act antagonistically through PbTpk2 to increase and decrease *FLO11* expression, respectively, to induce and repress filamentation. Considering that PbTupA interacts with PbTpk2, possibly via the second glutamine-rich N-terminal domain (Figs 2 and 5), but not PbTpk1 (Table 1), and that there are substantive differences in the sequences of the N-terminal domains in different fungal Tpk isoforms, this is suggestive of a role in defining the substrate specificity of these PKAs. Our studies show that PbTpk2 and PbTpk1 differ in their localization and function; in analogy to studies of the different Tpk proteins in *S*. *cerevisiae* that are differentially localized [70] and give rise to different phophoproteomes [98]. In support of this supposition, recent studies in *S*. *cerevisiae* indicated that the N-terminal domain regulates the function of Tpk2 [99]. Furthering this notion, distinct morphogenetic roles were demonstrated with chimeras created by exchanging N-terminal extensions of Tpk proteins from *C*. *albicans* [100]. Although our studies indicated that PbTupA interacts with the N-terminal domain of PbTpk2, but not with that of PbTpk1 or ScTpk2, this domain was not required for targeting PbTpk2 to the nucleus and both N-terminal truncated PbTpk2 and ScTpk2 functioned with PbTupA to induce hyperfilamentation. Consequently, the role of the N-terminal domain of Tpk2 remains elusive, but considering that this domain can also bind other proteins, such as AC, our expectation is that the specificity can be modulated by these additional interactions.

**Fig 7:**
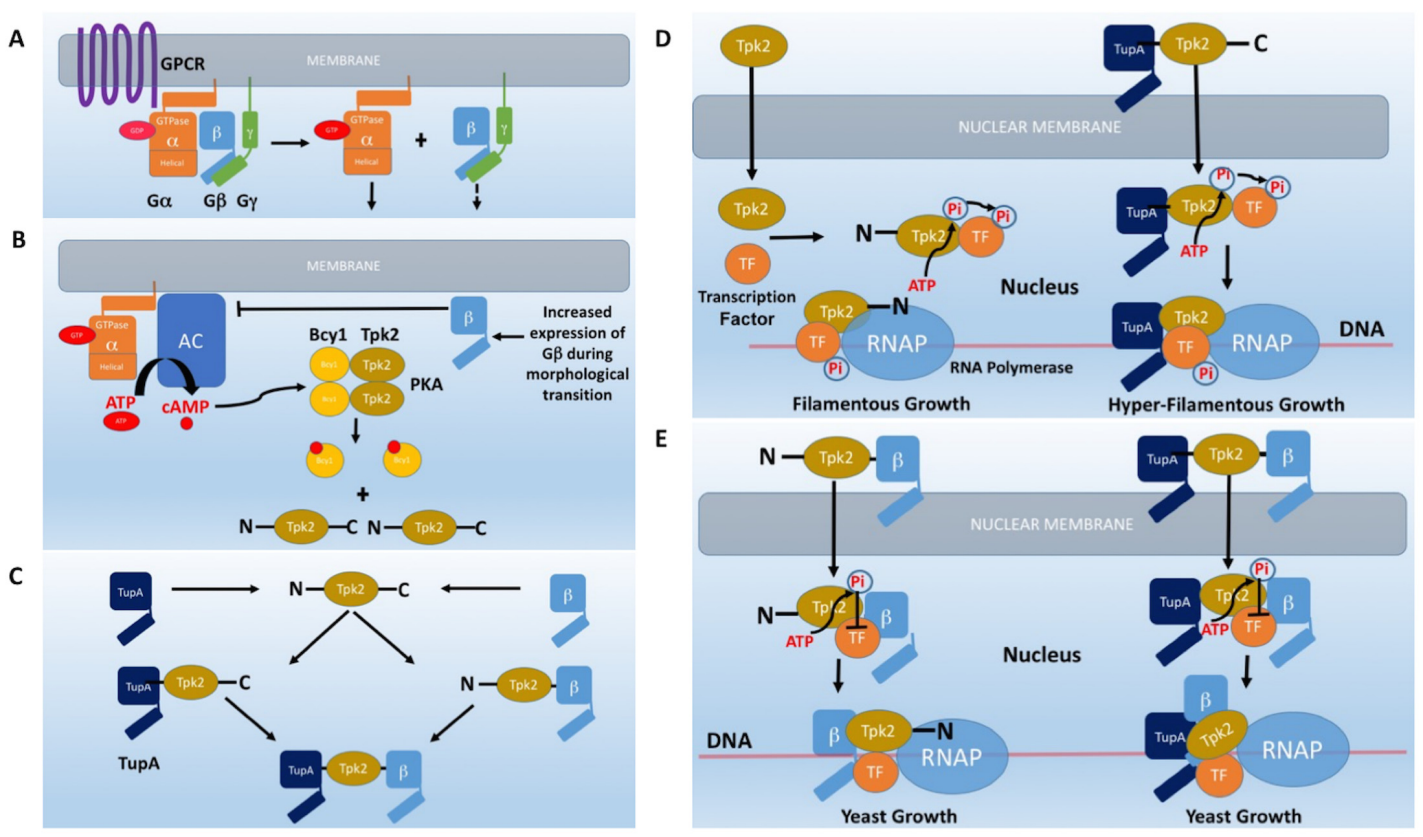
A model for the regulation of the fungal cAMP-signaling pathway by the Gβ and TupA proteins. The classical cAMP-signaling pathway, prevalent in dimorphic fungi, consists of a G-protein coupled receptor (GPCR), which is associated with a trimeric complex of the Gα, Gβ and Gγ proteins. **(A)** The GPCR responds to extracellular stimuli activating Gα, which exchanges GDP for GTP, and dissociates from the receptor and GβGγ complex. **(B)** The Gα-protein interacts with, and activates, adenylate cyclase (AC), which produces the signalling molecule cAMP. The Gβ protein can also interact with AC. Since the expression of Gβ is up-regulated during the morphological transition to the yeast form [19], this excess of free Gβ could act to curtail the signalling process. cAMP binds to Protein Kinase A (PKA), which is composed of two regulatory subunits (Bcy1) and two catalytic subunits (Tpk2). cAMP binds to the Bcy1 subunits inducing their release from the Tpk2 subunits. **(C)** Our data indicates that the WD/β-propeller proteins Gβ and TupA bind to Tpk2: Gβ binds to the C-terminal, catalytic domain, of Tpk2, whilst TupA, a transcriptional co-regulator, binds to the N-terminal domain of Tpk2. **(D)** Tpk2 is targeted to the nucleus, where it can interact with and phosphorylate transcription factors (TF) that form a transcriptional complex with RNA polymerase (RNAP) (lefthand figure). This complex is responsible for the expression of genes necessary for the morphological change (e.g. filamentous growth). TupA is also targeted to the nucleus, but may do so in complex with Tpk2, where it interacts with its target transcription factors (righthand figure). By binding to Tpk2, TupA may define those TFs to be phosphorylated and/or facilitate their phosphorylation; and/or facilitate specific interactions with the target DNA or transcription complex. Whatever the mechanism, the effect of TupA is to act as a strong inducer of the morphological change (e.g. hyperfilamentous growth). **(E)** The interaction of Gβ with Tpk2 facilitates its trafficking to the nucleus and, presumably via binding to the catalytic C-terminal domain, can suppress its kinase activity and the resulting filamentous gowth (lefthand figure). Although our data indicates that Gβ inhibits the kinase activity of Tpk2, the possibility that it alters the specificity of Tpk2 for target transcription factors cannot be excluded. We hypothesis that the TupA/Tpk2/TF complex is regulated by binding of Gβ, which inhibits the kinase activity of Tpk2 and/or alters its specificity (righthand figure). Whilst the binding of TupA and Gβ to Tpk2 could be mutually exclusive, which would provide a mechanism for the inhibitory action of Gβ, it seems likely that the complex is held together via multiple interactions (e.g. with TupA binding to both Tpk2 and a common TF) and that Gβ inhibits or alters the specificity for gene expression of the transcriptional complex. The phosphorylation of the TF is shown to occur before the interaction with the RNAP on the DNA for clarity, but phosphorylation could occur after the interaction.

In conclusion, our data suggests AC can act as a scaffold for the assembly of a multicomponent signaling-complex that can incorporate both up- and down-stream components of the signaling pathway; whilst Tpk2 can act as a scaffold for the assembly of a transcriptional complex that can be regulated by up-stream components of the signaling pathway. However, since AC interacts with Tpk2, there is a possibility that these complexes are one and the same, with the interesting possibility that they assemble in both the cytoplasm and nucleus. It will be important to elucidate how, when and where these complexes are assembled and disassembled in future studies. The Gβ-protein PbGpb1, which can bind both PbAC and PbTpk2, appears to act as a molecular switch that can turn-off the signaling pathway, by down-regulating the activity of both AC and Tpk2 (Fig 3). In contrast, PbTpk2 interacts with the regulatory-protein PbTupA, stimulating hyperfilamentous growth, an effect that PbGpb1 is capable of over-riding. Consequently, PbGpb1 and PbTupA act through PbTpk2 as antagonistic molecular switches of the cellular morphology (Fig 7). Considering that Tpk2 proteins can be physically associated with the genes that they regulate, and that Tup proteins interact with and regulates the RNA transcriptional machinery, suggests that Tpk2 acts as a scaffold for the assembly of a transcriptional complex (Fig 7). Further considering that PbTpk2 and PbTupA are targeted to the nucleus and that the inhibitory effect of PbGpb1 coincides with its accumulation in the nucleus, PbGpb1 might inhibit formation of this transcriptional complex. However, further studies will be needed to determine if PbGpb1 merely blocks assembly of the Tpk2-complex, effectively switching off Tpk2, or whether it simultaneously binds to the complex to regulate its function, by, for example, altering its specificity. Since other dimorphic fungi have homologues of *GPB1* and *TUPA*, this may represent a general mechanism for controlling the morphological switch that underpins the virulence of dimorphic fungi.

## Supporting Information

S1 Table Strains used in this study. (PDF)

S2 Table Plasmids used in this study. (PDF)

S3 Table Primers used for plasmid constructions used in this study and RT-PCR. (PDF)

S4 Table Primers used for gene cloning. (PDF)

S1 Fig Phylogenetic relationship of cAMP-dependent PKAs. The phylogenetic tree was constructed by Vector NTI alignX (Informax), which explains the relationship between Pb cAMP-dependent PKA catalytic subunit Tpk with other related organisms PKAs. The abbreviations are: Pb, *P*. *brasiliensis*; Sc, *S*. *cerevisiae*; Af, *A*. *fumigatus*; An, *A*. *nidulans*; Ca, *C*.*albicans*; Um, *Ustilago maydis*.
(PDF)

S2 Fig Comparative alignment of cAMP-dependent PKA catalytic subunits. The proteins were aligned by Vector NTI alignment (Informax). The abbreviations are: Pb, *P*. *brasiliensis*; Sc, *S*. *cerevisiae*; Af, *Aspergillus fumigatus*; An, *Aspergillus nidulans*; Ca, *Candida albicans*; Um, *Ustilago maydis*. The following symbols indicate conserved sequences. Note the lack of an N-terminal domain in the mammalian PKA (e.g. PKAC-alpha from rat).
(PDF)

S3 Fig The transformation of the diploid *S*. *cerevisiae TPK2Δ* mutant with *P*. *brasiliensis TPK1*. (A) *PbTPK1* does not complement the *S*. *cerevisiae TPK2Δ* strain to produce pseudohyphae. *P*. *brasiliensis TPK1-GFP* was transformed into the *S*. *cerevisiae TPK2Δ* mutant *XPY5a/α* and then the transformants were streaked on SLAD agar and an individual colony was observed under 20x magnification. The transformant was unable to produce pseudohyphae in response to a limited nitrogen supply. (B) PbTpk1 localizes to distinct sites in the cytoplasm of *S*. *cerevisiae* cells. Confocal microscopy of cells expressing *PbTpk1-GFP*, in which the nucleus was localized by staining with DAPI.
(PDF)

S4 Fig PbGpa1 does not block pseudohyphae formation. (A) *P*. *brasiliensis GPA1-GFP* was transformed with or without *PbTPK2* into the *S*. *cerevisiae MLY61a/α* (WT) and the *TPK2Δ* mutant *XPY5a/α* and then the transformants were streaked on SLAD agar and an individual colony was observed under 20x magnification. The *XPY5a/α/PbGPA1* transformant was unable to produce pseudohyphae but the *MLY61a/α/PbGPA1* transformant and the *XPY5a/α/PbTPK2/PbGPA1* co-transformant could both produce pseudohyphae in response to a limited nitrogen supply. PbGpa1 does not bind Tpk2 to block pseudohyphae formation. (B) Pan-cellular distribution of PbGpa1 in *S*. *cerevisiae* cells. Confocal microscopy of cells expressing *PbGPA1-GFP*, in which the nucleus was localized by staining with DAPI.
(PDF)

S5 Fig PbTupA is highly conserved with TupA from *P*. *marneffei* and *A*. *nidulans*. Alignment was done with Vector NTI 6.0. GenBank protein accession numbers are indicated as following the name of proteins. The predicted N-terminal coiled-coil domain and seven WD40 repeats are shown with black lines.
(PDF)

S6 Fig PbTupA induces hyperfilamentous growth that can be repressed by PbGpb1. The *S*. *cerevisiae* diploid strain *MLY61a/α* (WT) and its *TPK2Δ* mutant *XPY5a/α* were transformed with the *PbTUPA*, *ScTUP1*, *PbTPK2* and *PbGPB1* as indicated. To allow selection, generally, constructs for the expression of the PbTupA-mRFP, ScTup1-mRFP, PbTpk2-FL-mRFP and PbGpb1-GFP, PbTpk2^(226–583)-^GFP, PbTpk2^(226–583)-^GFP K301R and PbTpk2^(265–583)-^GFP fusion proteins were used and the transformants, selected on the basis of their fluorescence The cells were analysed for pseudohyphal growth in SLAD agar containing 50 μM (upper panel) or 200 μM (middle panel) ammonium sulphate, and for invasive growth into SD—ura agar medium (bottom panel). Single colonies from the agar plate were observed at 20x magnification in an Eclipse E-400 microscope (upper and middle panels; scale bar 50 μm) and in a Leica M165 FC stereo fluorescence microscope (bottom panel; scale bar 0.8 inches). WT cells expressing PbTupA were hyperfilamentous; whilst those expressing ScTup1 did not produce pseudohyphae. XPY cells expressing PbTupA produced few pseudohyphae; whilst those expressing PbTupA with PbTpk2, but not a kinase defective K301R derivative, were hyperfilamentous, indicating the requirement for a functional PKA. The co-expression of PbGpb1 with PbTupA repressed the filamentous growth of the *XPY/PbTPK2* but not the WT cells, indicating that PbGpb1 specifically inhibits PbTpk2.
(PDF)

S7 Fig PbTupA is localized in the nucleus. *P*. *brasiliensis TUPA-mRFP* was transformed into the *S*. *cerevisiae MLY61a/α* (WT) diploid strain and its *TPK2Δ* mutant *XPY5a/α* and then the transformants were streaked on SLAD agar and an individual colony was observed under 20x magnification. Confocal microscopy of cells expressing PbTupA-mRFP, in which the nucleus was localized by staining with DAPI.
(PDF)
