# Supplementary material for: A Gβ protein and the TupA Co-Regulator Bind to Protein Kinase A Tpk2 to Act as Antagonistic Molecular Switches of Fungal Morphological Changes
Source: PLoS One. 2015 Sep 3;10(9):e0136866. doi: 10.1371/journal.pone.0136866 (PMC4559445; doi:10.1371/journal.pone.0136866)
Supplement: S1 Table — (PDF) [file pone.0136866.s001.pdf]

S1 Table. Strains used in this study.

Strains	Description/genotype	Source/Reference
Paracoccioides brasiliensis Pb01	Dimorphic, yeast form-37˚C (Requires Cys or Met as supplement) and mycelial form-25˚C (No supplements required)	ATCC 90659
Saccharomyces cerevisiae AH109	MATa, *trp* 1-901, *leu*2-3, ura3-52, his3-200, gal4Δ, gal80Δ, LYS2 :: $GAL1_{UAS}$-$GAL1_{TATA}$ -HIS3, $GAL2_{UAS}$- $GAL2_{TATA}$-ADE2, URA3 :: $MEL1_{UAS}$-$MEL1_{TATA}$-lacZ	Clontech
S. cerevisiae SGY 446	MATα *tpk*1Δ::ADE8 *tpk*2-63(Ts) *tpk*3::TRP1 BCY1 ura3-52 his3 *leu*2-3,112 *trp*1 ade8; PKA complementation assay.	[63]
S. cerevisiae MLY5a/α	*ura*3-52/*ura*3-52 MATa/ α; wild type; PKA complementation assay.	[20]
S. cerevisiae XPY5a/α	Δ*tpk*2::G418/Δ*tpk*2::G418 ura3-52/ *ura*3-52 MATa/α; PKA complementation assay.	[20]
S. cerevisiae XPY95a/α	*flo8::HygB/flo8::HygB ura3-52/ura3-52 MATa/α*	[20]
S. cerevisiae XPY107a/α	*Flo11::HygB/fl11::HygB ura3-52/ura3-52 MATa/α*	[20]
Escherichia coli NovaBlue	K-12 strain with high transformation efficiency; blue/ white screening capability (with pGEM-T), *recA* and *endA* mutations resulting in high yields of quality plasmid DNA.	Novagen
E. coli BL21(DE3) STAR	RNaseE (*rne131*) mutant; general purpose expression host with reduced mRNA degradation.	Invitrogen
E. coli BL21(DE3) Codon plus	Deficient in *lon* and *ompT* proteases; allows expression of genes encoding tRNAs for rare argenine and proline codons.	Stratagene
E. coli BL21(DE3) PlysS	Deficient in *lon* and *ompT* proteases; high-stringency expression host.	Stratagene
Agrobacterium tumefaciens LBA1100	Strain used for conjugative transfer	
