# Supplementary material for: A Gβ protein and the TupA Co-Regulator Bind to Protein Kinase A Tpk2 to Act as Antagonistic Molecular Switches of Fungal Morphological Changes
Source: PLoS One. 2015 Sep 3;10(9):e0136866. doi: 10.1371/journal.pone.0136866 (PMC4559445; doi:10.1371/journal.pone.0136866)
Supplement: S2 Table — (PDF) [file pone.0136866.s002.pdf]

S2 Table. Plasmids used in this study

Plasmids	Descriptions/ Application	Source/reference
P426 MET25	Yeast constitutive expression vector; *MET25* promoter; yeast complementation assay; Amp^R	[62]
pGADT7 (AD fusion)	Vector with Gal-4 activation domain; expressed at high level from the constitutive *ADH1* promoter; Cloning vector for Two-hybrid assay; Amp^R	Clontech
pGBKT7 (BD)	Vector with Gal-4 binding domain; expressed at high level from the constitutive *ADH1* promoter; Cloning vector for Two-hybrid assay; Kan^R	Clontech
pGBKT7 Lamin	Vector with Gal-4 binding domain; expressed at high level from the constitutive *ADH1* promoter; negative control vector for Two-hybrid assay; Kan^R	Clontech
pGADT7 Tag	Vector with Gal-4 activation domain; expressed at high level from the constitutive *ADH1* promoter; Positive control vector for Two-hybrid assay, Amp^R	Clontech
pGBKT7 P^{53}	Vector with Gal-4 binding domain; expressed at high level from the constitutive *ADH1* promoter; positive vector for Two-hybrid assay; Kan^R	Clontech
pGEM-T Easy	Cloning vector compatible with α-complementation; high copy number plasmid with Ampicillin resistance (Amp^R); TA cloning	Novagen
pET21d (+)	T7 *lac* expression vector for C-terminal His_6-tagged proteins, Amp^R	Novagen
pGEX6p-1-3	26 kDa N-terminus Glutathione S-transferase (GST) tag; *tac* promoter; cleaved by PreScission Protease; Amp^R	GE Healthcare
pDNR-Lib	Pb01 cDNA library; Amp^R	Clontech, cDNA library construct [19]
pGADT7-Lib	Pb01 cDNA library; Amp^R	Clonetech, cDNA-library construct [19]
pCR35	Delivery of As*GPB1* and As*TUPA* to *P. brasiliensis*	
