# Supplementary material for: A Gβ protein and the TupA Co-Regulator Bind to Protein Kinase A Tpk2 to Act as Antagonistic Molecular Switches of Fungal Morphological Changes
Source: PLoS One. 2015 Sep 3;10(9):e0136866. doi: 10.1371/journal.pone.0136866 (PMC4559445; doi:10.1371/journal.pone.0136866)
Supplement: S3 Table — (PDF) [file pone.0136866.s003.pdf]

S3 Table. Primers used for plasmid constructions used in this study and RT-PCR.

Constructions	Primers
**For yeast 2-hybrid analyses**	
pADT7 *PbTPK2*$^{226-583}$	For *Eco*RI 5′ GAATTCATTCACAGCATCCACAGCAACAACAACAC 3′<br>Rev *Xho*I 5′ CTCGAGTCAAAAGTCCACGAAATAATCGCCATATGG 3′
pADT7 *PbTPK2*$^{1-270}$	For *Nco*I 5′ CCATGGAACGGGGTCTAGGCAATTTGCTGAAGAAG 3′ 1F<br>Rev *Bam*HI 5′ GGATCCTTACGAGTATTTGCCCTTTGTCTG CCGC3′ 1R
pADT7 *PbTPK2*$^{1-583}$	For *Nco*I 5′ CCATGGAACGGGGTCTAGGCAATTTGCTGAAGAAG 3′1F<br>Rev *Bam*HI 5′ GGATCCTTAAAAGTCCACGAAATAATCGCCATATGGATCATC 3′2R
pADT7 *PbTPK2*$^{265-583}$	For *Nco*I 5′ CCATGGAAACAAAGGGCAAATACTCGCTAGATGACTTTACG′ 3F<br>Rev *Bam*HI 5′ GGATCCTTAAAAGTCCACGAAATAATCGCCATATGGATC 3′2R
pBKT7 *PbTPK2*$^{226-583}$	For *Nco*I 5′ CCATGGGCCATTCACAGCATCCACAGCAACAACAAC 3′<br>Rev *Bam*HI 5′ GGATCCAAAGTCCACGAAATAATCGCCATATGGATC 3′
pBKT7 *PbTPK2*$^{1-270}$	For *Nco*I 5′ CCATGGAACGGGGTCTAGGCAATTTGCTGAAGAAG 3′ 1F<br>Rev *Bam*HI 5′ GGATCCTTACGAGTATTTGCCCTTTGTCTGCCGC 3′ 1R
pBKT7 *PbTPK2*$^{1-583}$	For *NcoI* 5′ CCATGGAACGGGGTCTAGGCAATTTGCTGAAGAAG 3′1F Rev<br>Rev *Bam*HI 5′ GGATCCTTAAAAGTCCACGAAATAATCGCCATATGGATC 3′ 2R
pBKT7 *PbTPK2*$^{265-583}$	For *NcoI* 5′ CCATGGAAACAAAGGGCAAATATCGCTAGATGACTTTACG 3′ 3F<br>Rev *BamHI* 5′ GGATCCTTAAAAGTCCACGAAATAATCGCCATATGGATC 3′2R
pADT7 Pb*TPK2*<br>& pBKT7 *TPK2*$^{1-174}$	For *Nco*I 5′ ATAATACCATGGAACGGGGTCTAGGCAATTTGCTG 3′<br>Rev *Bam*HI 3′ ATAATAGGATCCTTATTTGAAATGAGAGGGATCGCCCG 3′
pADT7 *PbGPG1*	For *Nco*1 5′ CCATGGAAGCCCCTGCCTACGAGCTTCGACCC 3′<br>Rev *Bam*H1 5′ GGATCCTTACATGATCATACAGCAGCCACCTGATTGTTGGGG 3′
pBKT7 *PbGPG1*	For *Nco*I 5′ CCATGGAAGCCCCTGCCTACGAGCTTCGACCC 3′<br>Rev *Bam*HI 5′ GGATCCTTACATGATCATACAGCAGCCACCTGATTGTTGGG 3′
pGADT7 *PbTUPA*<br>& pGBKT7 *PbTUPA*	For *Nco*I 5′ ATACTACCATGGGTATGTATAACCCACACCGTGGTATGGTTA 3′<br>Rev *Sma*I 5′ ATACTACCCGGGTGACCTTCTGGGATCCAATTG 3′
pGADT7 *ScTPK2*	For *Bam*HI 5′ GGATCCATGGAATTCGTTGCAGAAAGGGC 3′<br>Rev *Xho*I 5′ CTCGAGTTAGAAATCTTGAAAGTATTCAGCATATGGATCATC 3′
pGADT7 ScGPR1$^{1-961}$	For *Bam*HI 5′ CCGGATCCAGGAAAAACCTTGGAACTATTCATG 3′<br>Rev *Bam*HI 5′ CGGGATCCATTTTCAAACATCGCGATAC 3′

pGADT7 *ScGPA2*	For *Nde*I 5′ CGCTCATATGGGTCTCTGCGCATCTTCAGAAAAG 3′<br>Rev *Xho*I 5′ CGAGCTCGAGCATTGTAACACTCCAGAGTCTTTC 3′
pBKT7 *ScGPA2*	For *Bam*HI 5′ CCGGATCCTGGGTCTCTGCGCATCTTCA 3′<br>Rev *Bam*HI 5′ CCGGATCCGCTGTGCATTCATTGTAACAC 3′
pBKT7 *ScCYR1*$^{1-365}$	For *Nco*I 5′ ATGGCCATGGCATCAAAACCTGATACTGGTTCG 3′<br>Rev *Bam*HI 3′ TCGCGGATCCTGTCAGAGTTAGAATTTTCCGG 3′
pBKT7 *ScCYR1*$^{350-480}$	For *Nco*I 5′ CGAGACCATGGCTCCAGAATATAATGAGAACATTC 3′<br>Rev *Bam*HI 3′ GATTTGGATCCCCCTTTAGGTTTTGTTTTTTTCC 3′
pBKT7 *ScCYR1*$^{600-800}$	For *Nco*I 5′ CGATTCCATGGCGTACACTAACGCGTATATGGAG 3′<br>Rev *Bam*HI 3′ TCCAGGATCCTTAAATCTACGTGAACAAATTCG 3′
**For protein expression in yeast**	
p426 MET25-*GFP*	For *Eco*RI 5′ CATATGAATTCATGGTGAGCAAGGGCGAGGAGCTGTTC 3′<br>Rev *Sal*I 5′ ATATAGTCGACTTACTTGTACAGCTCGTCCATGCCGAGAGTG 3′
p426 MET25 *PbTPK2*$^{1-225}$-GFP	For *Bam*HI 5′ AATATGGATCCATGCGGGGTCTAGGCAATTTGCTGAAG3′<br>Rev *Eco*RI 5′ ATAATGAATTCTCGTTGTTGTGCGGCATGTAGGCCATC 3′
p426 MET25 Pb*TPK2*$^{226-583}$-GFP	For *Bam*HI 5′ CATATGGATCCATGCATTCACAGCATCCCAGCAACAACAAC 3′<br>Rev *Eco*RI 5′ CTCCTGAATTCAAAGTCCACGAAATAATCGCCATATGGATC 3'
p426 MET25 *PbGPB1-GFP*	For *Bam*HI 5′ ATACATGGATCCATGGCGGCCGATTTGAGCG 3′<br>Rev *Hind*III 5′ ATCTCTAAGCTTCCATGCCCAGACCTTGAGCAG 3′
p426 MET25 *PbTPK2*$^{1-583}$	For *Bam*HI 5′ ATTATAGGATCCATGCGGGGTCTAGGCAATTTG 3′<br>Rev *Eco*RI 5′ CTCCTGAATTCTTAAAAGTCCACGAAATAATCGCCATATG 3′
p426 MET25 *GFP*	For *Hind*III 5′ CATATAAGCTTATGGTGAGCAAGGGCGAGGAGC 3′<br>Rev *Sal*I 5′ ATATAATCGATTTACTTGTACAGCTCGTCCATGCCGAGAGTG 3′
p426 MET25 *PbTPK1*$^{135-560}$-*GFP*	For *Bam*H1 5′ ATAATAGGATCCATGGCCGCGGAAACGATAC 3′<br>Rev *Hind*III 5′ CTACTCAAGCTTAAAATCCGCAAACATAGCATCATGC 3′
pADT7 *PbTPK1*$^{135-560}$<br>& BKT7 *PbTPK1*$^{135-560}$	For *Nco*I 5′ ATAATACCATGGAAATGGCCGCGGAAACGATAC 3′<br>Rev *Bam*HI 5′ ATAATCGGATCCTCAAAAATCCGCAAACATAGCATCATG 3′
P426 MET25-*mRFP*	For *Hind*III 5′ CTGACAAGCTTATGGCCTCCTCCGAGGACGTCATCAAGGAG 3'<br>Rev *Sal*I 5′ ATAATGTCGACTTAGGCGCCGGTGGAGTGGCGGCCCTC 3′

P426 MET25-*PbTUPA-mRFP*	For *Spe*I 5′ CTCCTCACTAGTATGTATAACCCACACCGTGGTATG 3′<br>Rev *Hind*III 5′ CTACTAAAGCTTCCTTCTGGGATCCCAATTGGAATAGC 3′
p426 MET25-*ScTUP1*-mRFP	For *Sma*I 5′ CCCGGGATGACTGCCAGCGTTTCGAATAC 3′<br>Rev *Hind*III 5′ AAGCTTATTTGGCGCTATTTTTTTATACTTCCAAATCCTTGC 3′
p426 MET25 *PbTPK2*$^{(1-583)}$- *mRFP*	For *Bam*HI 5′ AATATGGATCCATGCGGGGTCTAGGCAATTTGCTGAAG 3′<br>Rev *Eco*RI 5′ CTCCTGAATTCAAAGTCCACGAAATAATCGCCATATGGATCATC 3′
P426MET25 *PbTPK2*$^{(265-583)}$*-GFP*	For BamHI 5′GGATCCATGACAAAGGGCAAATACTCGCTAGATGACTTTACG 3′<br>Rev *Eco*RI 5′ CTCCTGAATTCAAAGTCCACGAAATAATCGCCATATGGATCATC 3′
**For adenylate cyclase (Cyr1) overexpression in *E. coli***	
pGEX6p-3 *PbCYR1*$^{453-678}$	For *Bam*H1 5′ AAGGATGGATCCGATAAAACCCATCAGGAT 3′<br>Rev *Not*I 5′ CATATCGCGGCCGCTTAGTGGCTAAACTTTTGGTTCTCGTTG 3′
**For G protein overexpression in *E. coli***	
pGEX6p-3 Pb*GPB1*	For *Bam*HI 5′ ATACATGGATCCATGGCGGCCGATTTGAGCGGCG 3′<br>Rev *Not*I 5′ ATATCTGCGGCCGCCTACCATGCCCAGACCTTG 3′
pGEX6p-3 *PbGPG1*	For *Bam*HI 5′ ATCTATGGATCCATGGCCCCTGCCTACGAGCTTCG 3′<br>Rev *Not*I 5′ ATCTATGGATCCATGGCCCCTGCCTACGAGCTTCG 3′
**For *Pb* Tpk2 protein overexpression in *E. coli***	
pGEX6p-3 *PbTPK2*$^{1-225}$	For *Bam*HI 5′ ATTATAGGATCCATGCGGGGTCTAGGCAATTTGCTGAAG 3′<br>Rev *Sal*I 5′ ATAATAGTCGACTTATCGTTGTTGGCGGCATGTAGGCC 3′
pET21d(+)*PbTPK2*$^{226-583}$	For *Nco*I 5′ CCATGGGACATTCACAGCATCCACAGCAACAAC 3′<br>Rev *Xho*I 5′ CTCGAGAAAGTCCACGAAATAATCGCCATATG 3′
**For TupA overexpression in *E. coli***	
pET21d(+) *PbTUPA*	For *Nhe*I 5′ ATACTAGCTAGCTATAACCCACACCGTGGTATGGTTAC 3′<br>Rev *Xho*I 5′ ATAATACTGGAGCCTTCTGGGATCCCAATTGGAATAGC 3′
**Primers used for RT-PCR for *FLO11***	For 5′ GTTATTACCACTGAGTCATCTGTTG 3′<br>Rev 5′ GTTGTAGCTAGTTGGGATGTAG 3′

**Primers used for RT-PCR for Actin**	For 5′ CGTGCTGTCTTCCCATCTATC 3′<br>Rev 5′ GTAGAAGGTATGATGCCAGATC 3′
**Primers for Mutagenesis work**	
p426MET25 and pGBKT7 *PbTPK2*	For 5′ CCAGCGCTTCTATGCCATCACGGTGCTGAAGAAAG 3′<br>Rev 5′ CTTTCTTCAGCACCGTGATGGCATAGAAGCGCTGG 3′
p426 MET25-*PbGPB1* S109R-*GFP*	For 5' CCATCCCCCTAAGATCGAGATGGGTGATGACTTGCG 3′<br>Rev 5' CGCAAGTCATCACCCATCTCGATCTTAGGGGGATGG 3′
p426 MET25-*PbGPB1* S151R-*GFP*	For 5′ TGTCGCACGAGAATTGCGCGGCCACACCG 3′<br>Rev 5′ CGGTGTGGCCGCGCAATTCTCGTGCGACA 3′
**Primers for amplification of Pb01 antisenses**<br>**A1Gpb1**<br><br>**A2Gpb1**<br><br>**A3Gpb1**<br><br>**A1TupA**<br><br>**A2TupA**<br><br>**A3TupA**	 <br>For *Xho*I 5′ gcgcCTCGAGACAATTGCGTCTGGCTAGGT 3′<br>Rev *Asc*I 5′ gcgcGGCGCGCCCCACCCGTCTTATTTGAGGA 3′<br>For *Xho*I 5′ gcgcCTCGAGCAACTGCTGGAGTCCCAAGT 3′<br>Rev *Asc*I 5′ gcgcGGCGCGCCAGAGGGTGGTCAACTGGATG 3′<br>For *Xho*I 5′ gcgcCTCGAGGCGGTAACTATGTCGCCTGT 3′<br>Rev *Asc*I 5′ gcgcGGCGCGCCCGCGGATATCCCAGAGTTTA 3′<br>For *Xho*I 5′ gcgcCTCGAGCGCAGCTCTCTTCCTGTCTT 3′<br>Rev *Asc*I 5′ gcgcGGCGCGCCTGGCCAGTGAGTTGATGTTC 3′<br>For *Xho*I 5′ gcgcCTCGAGCATCTCCCCCAGTCTCACAT 3′<br>Rev *Asc*I 5′ gcgcGGCGCGCCACCTTGCTGTGGTTGAGGAG 3′<br>For XhoI 5′ gcgcCTCGAGCAACATCGAAGATGGCGTTA 3′<br>Rev AscI 5′ gcgcGGCGCGCCAGGGACCATCCCTCTAGGC 3′
**Primers used for RT-PCR**<br>Pb*GPB1*<br><br>Pb*TUPA*	 For 5′ ACACCACCTGCCGTCTATTC 3′<br>Rev 5′ CAGCAAACAAAAGTCGACCA 3′<br>For 5′ GGATCGTGGCGTACAGTTCT 3′<br>Rev 5′ CCAGATTCTAGCACGCATGT 3′
