# Supplementary material for: A Gβ protein and the TupA Co-Regulator Bind to Protein Kinase A Tpk2 to Act as Antagonistic Molecular Switches of Fungal Morphological Changes
Source: PLoS One. 2015 Sep 3;10(9):e0136866. doi: 10.1371/journal.pone.0136866 (PMC4559445; doi:10.1371/journal.pone.0136866)
Supplement: S4 Table — (PDF) [file pone.0136866.s004.pdf]

S4 Table. Primers used for gene cloning.

Primers	Sequences
PbTPK2	
PbTPK-F1	CATCCRTTYHTBATTMGAATGTGGGG
PbTPK-F2	TGGGTACTGGHTCYTTTGGNMGNGT
PbTPK-F3	CACGTTCCAGGATGTGAAGAATC
PbTPK-F4	GTTTACTGAGGAAATCGCAGCGCTTC
PbTPK-F5	TGCAAGCCAGTTTGATAGGTATC
PbTPK2-F6	ATCCACCCCTCAACGATGAAGTGG
PbTPK2-F7	GATGCGCCGTATATTCCTCCGGTGAAGGGA
PbTPK2-F8	GACGGAGCCGTATGGGGGTAGTGGGGATGA
PbTPK2-F9	AGACTAACAGCGGGGTCTGTGA
PbTPK2-F10	CTGAGACGGGTCTATGTTCGATGG
PbTPK-F40	GCTGTTTAGTTTACTGAGGAAATCGCAGCGCTTC
PbTPK-R1	ACBTMTTTKGCRAABCCRAARTC
PbTPK-R2	ATSARMAYMCCNARVSWCCACCARTC
PbTPK-R3	CTTCAAATGTCCATATCGATC
PbTPK-R4	CTAAAAGTAGATTCTCTGGCTTTAGGTCTCTG
PbTPK-R5	GTGATGGTCATGTAGATATTCCAACGCCAACG
PbTPK-R6	CTAAAAGTAGATTCTCTGGCTTTAGGTCTCGG
PbTPK2-R7	TACCTGGAACGTGCCCCATAGCG
PbTPK2-R8	TAGGAATGGATGTTTAACCCGCTGCAGC
PbTPK-R9	TAATGTTCTTAATACTGGCGACATTTTGGGAGTG
PbTPK2-R10	ATGTCGCGATTGAACCAGGTGTACTCGC
PbTPK-R11	CTCCGTCTAGATATTGGCATAGGC
PbTPK2-R12	GGGTGAGGTAAACGCAGGGTGCTGGAGCA
PbTPK2-R13	CTTCAGCAAATTGCCTAGACCCCGCATGGA
PbTPK2-R14	GGGACTACGGCAAGGCCTGTGGGTGGATGA
PbTPK2-R15	ATGATGCTGAGGCCCGACGCCACTAGATAG
PbTPK-R16	GTAGAATGGAATTGAACCTGCAGACACTCA
PbTPK2-R17	GTCCACGAAATAATCGCCATATGGATCATCC
PbTPK2-R18	CCCTAAGGGTATCAGGCGTGCCA
PbTPK-R40	CGATCTAAAAGTAGATTCTCTGGCTTTAGGTCTCTG
PbTUPA	
cPbTUPA-F1	ACTTCTTACCTTAGGCCAGCCTCGCCCTCA
cPbTUPA-F2	ACACCCGATGGACGCTGGGTTATGAGTGGA
cPbTUPA-F3	ATCGTGGCGTACAGTTCTGGGATCCAGCGA
cPbTUPA-F4	CCCGTTCAACCCAGCCTACCAACTACCGCA
cPbTUPA-R1	ATTATATGATGACGGCGGTGATAACGAGGA
cPbTUPA-R2	ATAGCTGTAAATCCCATGTCGAGAGTTAGG
cPbTUPA-R3	CCTTCCTCTGGCAAAGTTACGTGTTGCGCA
cPbTUPA-R7	CTGATCATCTCCATCTCCTGGAGTTGGCCA

PbTUPA-F1	MGSACYTTTGARGGNCAYAAR
PbTUPA-R1	CCARATTCKNGCNCKCATRTC
PbTUPA-R2	GTCGCTGGATCCCAGAACTGTACGCCACGA
PbTUPA-R3	CACTCATAACCCAGCGTCCATCGGGTGTCA
PbTUPA-R4	GGATATCCCAGACCTGTAGGTGGA
PbTUPA-R5	GGCAACTTGTCAGGATCCAGGTC
PbTUPA-R6	GAGTGCGGATTTCAGCCTCG
ScCYR1	
ScAC-BD-F1(NcoI)	ATGGCCATGGCATCAAAACCTGATACTGGTTCG
ScAC-BD-F350(NcoI)	CGAGACCATGGCTCCAGAATATAATGAGAACATTC
ScAC-BD-F600(NcoI)	CGATTCCATGGCGTACACTAACGCGTATATGGAG
ScAC-BD-R365(BamHI)	TCGCGGATCCTGTCAGAGTTAGAATTTTCCGG
ScAC-BD-R480(BamHI)	GATTTGGATCCCCCTTTAGGTTTTGTTTTTTTCC
ScAC-BD-R800(BamHI)	TCCAGGATCCTTAAATCTACGTGAACAAATTCG
ScGPR1	
ScGPR1-F1	CGGGATCCGAAGTGTGACGAATAAAGC
ScGPR1-F2	GCTGGGACATCAGACTTTCTCTTGCC
ScGPR1-F3	CGGGATCCATATGATAACTGAGGGATTTCCCCCG
ScGPR1-F4	CCGGATCCGTGAAAGTAAAAGAATTAAAGCGC
ScGPR1-F5	CCGGATCCAGGAAAAACCTTGGAACTATTCATG
ScGPR1-R1	CGGGATCCATTTTCAAACATCGCGATAC
ScGPR1-R2	GTTACTAGTTATGTCGCTGTTATCG
ScGPR1-R3	CCGGATCCTTAGATTCTTTTGAATTTGTGCC
ScGPA2	
ScGPA2-F1	CCGGATCCTGGGTCTCTGCGCATCTTCA
ScGPA2-F2	GGGTCTCTGCGCATCTTGAG
ScGPA2-F3	CCGGATCCGTATGGGTCTCTGCGCATCTTCAG
ScGPA2-F4	ATCATGGGTCTCTGCGCATC
ScGPA2-R1	CCGGATCCGCTGTGCATTCATTGTAACAC
ScGPA2-R2	AACGCGAGAAGAGGCATGCAG

Notes: primers sequences are 5'→3'; restriction sites are underlined; A+C+G=V, T+C+G=B, A+T+G=D, A+T+C=H, A+T=W, C+G=S, T+G=K, A+C=M, C+T=Y, A+G=R, A+T+C+G=N
