# Supplementary material for: A Gβ protein and the TupA Co-Regulator Bind to Protein Kinase A Tpk2 to Act as Antagonistic Molecular Switches of Fungal Morphological Changes
Source: PLoS One. 2015 Sep 3;10(9):e0136866. doi: 10.1371/journal.pone.0136866 (PMC4559445; doi:10.1371/journal.pone.0136866)
Supplement: S1 Fig — The phylogenetic tree was constructed by Vector NTI alignX (Informax), which explains the relationship between Pb cAMP-dependent PKA catalytic subunit Tpk with other related organisms PKAs. The abbreviations are: Pb, P. brasiliensis; Sc, S. cerevisiae; Af, A. fumigatus; An, A. nidulans; Ca, C.albicans; Um, Ustilago maydis. (PDF) [file pone.0136866.s005.pdf]

S1 Fig. Phylogenetic relationship of cAMP-dependent PKAs. The phylogenetic tree was constructed by Vector NTI alignX (Informax), which explains the relationship between *Pb* cAMP-dependent PKA catalytic subunit Tpk with other related organisms PKAs. The abbreviations are: Pb, *P. brasiliensis*; Sc, *S. cerevisiae*; Af, *A. fumigatus;* An*, A. nidulans;* Ca, *C. albicans;* Um*, Ustilago maydis*.

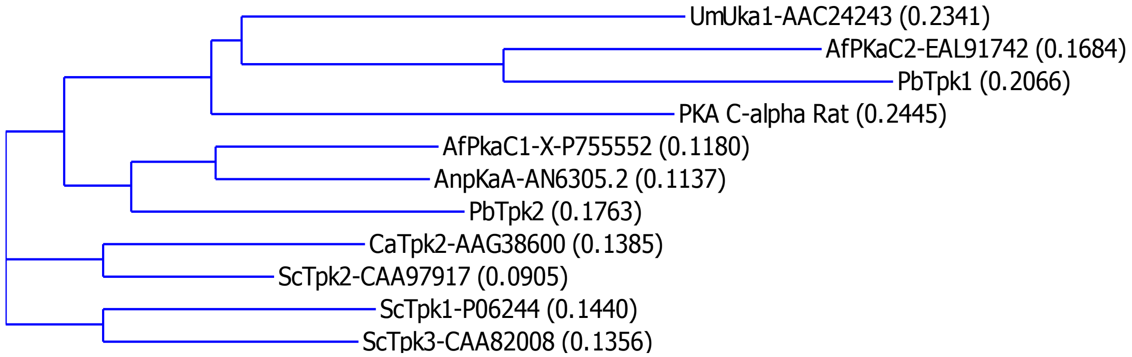

Tpk2 homology with other PKAs

S.No	Organism/protein	Identity with *Pb*Tpk2 (226-583) (C-terminus)	Identity with *Pb*Tpk2 Full-length(1-583)
1	*S. cerevisiae* Tpk1	54%	37.7%
2	*S. cerevisiae* Tpk2	65%	43.4%
3	*S. cerevisiae* Tpk3	56.4%	39.5%
4	*C. albicans* Tpk2	65%	43.4%
5	*A. fumigatus* pkaC1	60.6%	57.7%
6	*A. nidulans* AnpkaA	58.1%	53.5%
7	*U. maydis* uka1	42%	28.6%
8	*U. maydis* adr-1	53.9%	39.4%
9	*A. fumigatus* AfpkaC2-EAL9142	40.6%	27.8%
10	*P. brasiliensis* Tpk1	28.1%	27.8%

Tpk1 homology with other PKAs

S.No	Organisms	Identity with *Pb*Tpk1
1	*S. cerevisiae* Tpk1	27.2%
2	*S. cerevisiae* Tpk2	27.3%
3	*S. cerevisiae* Tpk3	27.9%
4	*C. albicans* Tpk2	29.2%
5	*A. fumigatus* AfpkaC1	31.5%
7	*A. nidulans* AnpkaA	30.4%
8	*A. fumigatus* AfpkaC2-EAL91742	43.6%
9	*P. brasiliensis* Tpk2	31.5%
10	*U. maydis* adr1	27.6%
11	*U. maydis* uka1	29%
