# Supplementary material for: A Gβ protein and the TupA Co-Regulator Bind to Protein Kinase A Tpk2 to Act as Antagonistic Molecular Switches of Fungal Morphological Changes
Source: PLoS One. 2015 Sep 3;10(9):e0136866. doi: 10.1371/journal.pone.0136866 (PMC4559445; doi:10.1371/journal.pone.0136866)
Supplement: S2 Fig — The proteins were aligned by Vector NTI alignment (Informax). The abbreviations are: Pb, P. brasiliensis; Sc, S. cerevisiae; Af, Aspergillus fumigatus; An, Aspergillus nidulans; Ca, Candida albicans; Um, Ustilago maydis. The following symbols indicate conserved sequences. Note the lack of an N-terminal domain in the mammalian PKA (e.g. PKAC-alpha from rat). (PDF) [file pone.0136866.s006.pdf]

S2 Fig. Comparative alignment of cAMP-dependent PKA catalytic subunits. The proteins were aligned by Vector NTI alignment (Informax). The abbreviations are: Pb, *P. brasiliensis*; Sc, *S. cerevisiae*; Af, *Aspergillus fumigatus;* An, *Aspergillus nidulans;* Ca, *Candida albicans;* Um, *Ustilago maydis*. The following symbols indicate conserved sequences. Note the lack of an N-terminal domain in the mammalian PKA (e.g. PKAC-alpha from rat).

Nucleotide binding region 'GTGSFG'

Catalytic loop 'RDLKPEN'

Mg^{2+} binding site 'TDFGFAK'

Autophosphorylation site 'T'

Regulatory and catalytic subunit association site 'H'

PKA function abolished by mutating 'K'

Glutamine 'Q' residues (175-186 and 229-254)

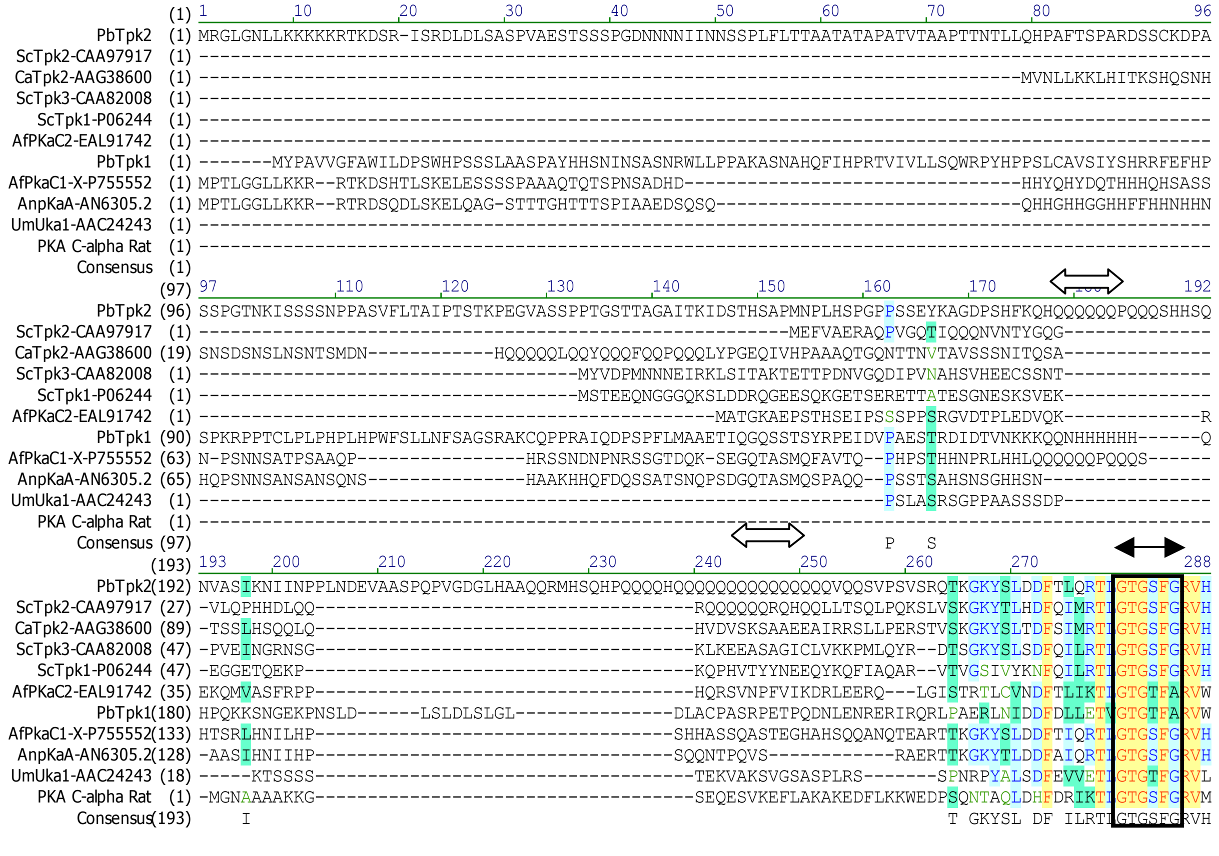

```
(289)                 289        300        310        320        330        340        350        360        370           384
PbTpk2(288)           LVQSRH-----NQRFYAIKVLKKAQVVKMKQVEHTNDE-RRMLQRVKHPFLITLWGTFQDVKNLYMVMDFVEGGELFSLLRKSQRFPNPVAKFYAA
ScTpk2-CAA97917 (86)  LVRSVH-----NGRYYAIKVLKKQQVVKMKQVEHTNDE-RRMLKLVEHPFLIRMWGTFQDARNIFMVMDYIEGGELFSLLRKSQRFPNPVAKFYAA
CaTpk2-AAG38600(148)  LVRSVH-----NGRYYAIKVLKKHQVVKMKQVEHTNDE-RRMLKLVEHPFLIRMWGTFQDSKNLFMVMDYIEGGELFSLLRKSQRFPNPVAKFYAA
ScTpk3-CAA82008(104)  LIRSNH-----NGRFYALKTLKKHTIVKLKQVEHTNDE-RRMLSIVSHPFIIRMWGTFQDSQQVFMVMDYIEGGELFSLLRKSQRFPNPVAKFYAA
ScTpk1-P06244(103)    LIRSRH-----NGRYYAMKVLKKEIVVRLKQVEHTNDE-RLMLSIVTHPFIIRMWGTFQDAQQIFMIMDYIEGGELFSLLRKSQRFPNPVAKFYAA
AfPKaC2-EAL91742 (92) LAKFKDETIR-RDNVYALKVLRKADVIKLKQVEHVRNERKALAAVAGHPFITTLIASFSDEQSLYMLLDYCPGGEIFSYLRRARRFNETTSRFYAA
PbTpk1(255)           LARLVKRKEG-QDGVYAIKILHKADVIKLKQVEHVRNEVRTLAAVSGHPFIISLISTFSDDQNLYMLLDYCPGGEVFTFLRRAHRFSERTSQFYAA
AfPkaC1-X-P755552(195) LVQSKH-----NHRFYAIKVLKKAQVVKMKQVEHTNDE-RRMLNRVRHPFLITLWGTWQDARNLYMVMDFVEGGELFSLLRKSQRFPNPVAKFYAA
AnpKaA-AN6305.2(177)  LVQSKH-----NHRYYAIKVLKKAQVVKMKQIEHTNDE-RRMLNRVRHPFLVTLWGTWQDARNLYMVMDFVEGGELFSLLRKSQRFPNPVAKFYAA
UmUka1-AAC24243 (66)  LVRLKDRDVADRSAYFALKVLAKTDVIKLKQVSHINSE-RCILTKVDHPFLVNMIASFQDSKNCYMLMEYVVGGEIFSYLRRAGHFSADVARFYIS
PKA C-alpha Rat (60)  LVKHKE-----SGNHYAMKILDKQKVVKLKQIEHTLNE-KRILQAVNFPFLVKLEFSFKDNSNLYMVMEYVPGGEMFSHLRRIGRFSEPHARFYAA
Consensus(289)        LVRSKH     N RYYAIKVLKK  VVKLKQVEHTNDE RRML IV HPFLI LWGTFQDAKNLYMVMDYVEGGELFSLLRKSQRFPNPVAKFYAA

(385)                 385 390        400        410        420        430        440        450        460        470       480
PbTpk2(378)           EVTLALEYLHDHH-IIYRDLKPENILLDRYGHLKITDFGFAKEVKD-ITWTLCGTPDYLAPEVVSSKGYNMSVDWWSLGILIFEMLCGFTPFWDGG
ScTpk2-CAA97917(176)  EVILALEYLHAHN-IIYRDLKPENILLDRNGHIKITDFGFAKEVQT-VTWTLCGTPDYIAPEVITTKPYNKSVDWWSLGVLIYEMLAGYTPFYDT-
CaTpk2-AAG38600(238)  EVTLALEYLHSHD-IIYRDLKPENILLDRNGHIKITDFGFAKEVST-VTWTLCGTPDYIAPEVITTKPYNKSVDWWSLGVLIFEMLAGYTPFYDS-
ScTpk3-CAA82008(194)  EVCLALEYLHSKD-IIYRDLKPENILLDKNGHIKITDFGFAKYVPD-VTYTLCGTPDYIAPEVVSTKPYNKSVDWWSFGVLIYEMLAGYTPFYNS-
ScTpk1-P06244(193)    EVCLALEYLHSKD-IIYRDLKPENILLDKNGHIKITDFGFAKYVPD-VTYTLCGTPDYIAPEVVSTKPYNKSIDWWSFGILIYEMLAGYTPFYDS-
AfPKaC2-EAL91742(187) EITLTIEYLHDVEGIVYRDLKPENILLDADGHIKIVDFGFAKQINDRETYTLCGTPEYLAPEVIHNSGHGLAVDWWALGILIYEFLVGQPPFWDQ-
PbTpk1(350)           EIVLILEFLHEVHGVVYRDLKPENILLDAEGHLKIVDFGFAKQLWSRETYTLCGTPEYLAPEVIHNKGHGLAVDWWALGVLIYEFIVGQPPFWDQ-
AfPkaC1-X-P755552(285) EVTLALEYLHSQQ-IIYRDLKPENLLLDRHGHLKITDFGFAKEVPD-ITWTLCGTPDYLAPEVVSSKGYNKSVDWWSLGILIFEMLCGFTPFWDSG
AnpKaA-AN6305.2(267)  EVTLALEYLHSLN-IIYRDLKPENLLLDRHGHLKITDFGFAKEVPD-ITWTLCGTPDYLAPEVVASKGYNKSVDWWSLGILIFEMLCGFTPFWDQG
UmUka1-AAC24243(161)  TIVLAIEYLHSNK-VVYRDLKPENLLIDSNGYTKITDFGFAKEVED-RTWTLCGTPEYLAPEIIQCSGHGSAVDWWSLGILLFEMLAGYPPFYDP-
PKA C-alpha Rat(150)  QIVLTFEYLHSLD-LIYRDLKPENLLIDQQGYIQVTDFGFAKRVKG-RTWTLCGTPEYLAPEIILSKGYNKAVDWWALGVLIYEMAAGYPPFFAD-
Consensus(385)        EV LALEYLHS   IIYRDLKPENILLDRNGHIKITDFGFAKEV D VTWTLCGTPDYLAPEVISSKGYNKSVDWWSLGILIYEMLAGYTPFWD

(481)                 481      490        500        510        520        530        540        550        560             576
PbTpk2(472)           SPMKIYENIIKCRVKYPSYMHPDAQDLLSQLITPDLTVRLGNLHGGSKDVKDHPWFAEVTWDRLARKDIDAPYIPPVKGGQGDASQFDRYPEETE-
ScTpk2-CAA97917(269)  TPMKTYEKILQGKVVYPPYFHPDVVDLLSKLITADLTRRIGNLQSGSRDIKAHPWFSEVVWERLLAKDIETPYEPPITSGIGDTSLFDQYPEEQL-
CaTpk2-AAG38600(331)  TPMKTYEKILAGKIHYPSFFQPDVIDLLTKLITADLTRRLGNLINGPADIRNHPWFSEVVWEKLLAKDIETPYEPPITAGVGDSSLFDHYPEEQL-
ScTpk3-CAA82008(287)  NTMKTYENILNAELKFPPFFHPDAQDLLKKLITRDLSERLGNLQNGSEDVKNHPWFNEVIWEKLLARYIETPYEPPIQQGQGDTSQFDRYPEEEF-
ScTpk1-P06244(286)    NTMKTYEKILNAELRFPPFFNEDVKDLLSRLITRDLSQRLGNLQNGTEDVKNHPWFKEVVWEKLLSRNIETPYEPPIQQGQGDTSQFDKYPEEDI-
AfPKaC2-EAL91742(282) NPMRIYEQIVEGRLRFPPNMSPAAQNIISCLCKTNPSERLGHISGGSARVKAHPFFEDIDWDDLFHRRMKGPIIPRVD-HPADTGNFEDYPDV---
PbTpk1(445)           NPMCIYEQIVQGRLRFPVNMPSTARDIVTQLCTTNPSERLGYIKGGAARVKQHPFFKDINWDDIYFRRTKGPIVPRVD-SPTDTGNFEEYPDEP--
AfPkaC1-X-P755552(379) SPVKIYENILRGRVKYPPYLHPDAVDLLSQLITADLTKRLGNLHGGSEDVKNHPWFAEVTWDRLARKDIDAPYVPPIRGGQGDASQYDRYPEETE-
AnpKaA-AN6305.2(361)  SPVKIYQNILAGRIKFPPYLHPDAVDLLSRLITSDLTKRLGNLHGGPDDIKNHPWFAEVTWDRLLRKEIDAPYVPPIRGGQGDASQYENYQEESE-
UmUka1-AAC24243(254)  NPILIYEKILAGNLVFPEEIDPLSRDLISSLLTADRSRRLGNLRGGANDVKNHPWFHGVDWKALQEGRILPPIVPYLG-RPGDTSNFSKYEPARPS
PKA C-alpha Rat(243)  QPIQIYEKIVSGKVRFPSHFSSDLKDLLRNLLQVDLTKRFGNLKNGVNDIKNHKWFATTDWIAIYQRKVEAPFIPKFK-GPGDTSNFDDYEEEE--
Consensus(481)        NPMKIYENIL GRLKFPPYM PDA DLLS LIT DLTKRLGNL GGS DVKNHPWFAEV WDRL  K IEAPYVPPI  G GDTSQFD YPEE

(505)                 505 510        520        530        540        550        560        570        580        590       600
PbTpk2(496)           QDLLSQLITPDLTVRLGNLHGGSKDVKDHPWFAEVTWDRLARKDIDAPYIPPVKGGQGDASQFDRYPEETE-------PYGGSGDDPYGDYFVDF-
ScTpk2-CAA97917(293)  VDLLSKLITADLTRRIGNLQSGSRDIKAHPWFSEVVWERLLAKDIETPYEPPITSGIGDTSLFDQYPEEQL-------DYGIQGDDPYAEYFQDF-
CaTpk2-AAG38600(355)  IDLLTKLITADLTRRLGNLINGPADIRNHPWFSEVVWEKLLAKDIETPYEPPITAGVGDSSLFDHYPEEQL-------DYGSQGEDPYASYFLDF-
ScTpk3-CAA82008(311)  QDLLKKLITRDLSERLGNLQNGSEDVKNHPWFNEVIWEKLLARYIETPYEPPIQQGQGDTSQFDRYPEEEF-------NYGIQGEDPYMDLMKEF-
ScTpk1-P06244(310)    KDLLSRLITRDLSQRLGNLQNGTEDVKNHPWFKEVVWEKLLSRNIETPYEPPIQQGQGDTSQFDKYPEEDI-------NYGVQGEDPYADLFRDF-
AfPKaC2-EAL91742(306) QNIISCLCKTNPSERLGHISGGSARVKAHPFFEDIDWDDLFHRRMKGPIIPRVD-HPADTGNFEDYPDV---DVKGQAIYTDDMKKKYEAL-----
PbTpk1(469)           RDIVTQLCTTNPSERLGYIKGGAARVKQHPFFKDINWDDIYFRRTKGPIVPRVD-SPTDTGNFEEYPDEP--DSSLLTPYTKEMREKHDAMFADF-
AfPkaC1-X-P755552(403) VDLLSQLITADLTKRLGNLHGGSEDVKNHPWFAEVTWDRLARKDIDAPYVPPIRGGQGDASQYDRYPEETE-------QYGQQGEDVHGHLFPDF-
AnpKaA-AN6305.2(385)  VDLLSRLITSDLTKRLGNLHGGPDDIKNHPWFAEVTWDRLLRKEIDAPYVPPIRGGQGDASQYENYQEESE-------PYGQAGEDPHGHLFPDF-
UmUka1-AAC24243(278)  RDLISSLLTADRSRRLGNLRGGANDVKNHPWFHGVDWKALQEGRILPPIVPYLG-RPGDTSNFSKYEPARPSAMPGLYGADSGHHDLYADLFPDF-
PKA C-alpha Rat(267)  KDLLRNLLQVDLTKRFGNLKNGVNDIKNHKWFATTDWIAIYQRKVEAPFIPKFK-GPGDTSNFDDYEEEE---------IRVSINEKCGKEFTEF-
Consensus(505)         DLLS LIT DLTKRLGNL GGS DVKNHPWFAEV WDRL  K IEAPYVPPI  G GDTSQFD YPEE          YG  GEDPYA LF DF
```
