# Supplementary material for: A Gβ protein and the TupA Co-Regulator Bind to Protein Kinase A Tpk2 to Act as Antagonistic Molecular Switches of Fungal Morphological Changes
Source: PLoS One. 2015 Sep 3;10(9):e0136866. doi: 10.1371/journal.pone.0136866 (PMC4559445; doi:10.1371/journal.pone.0136866)
Supplement: S3 Fig — (A) PbTPK1 does not complement the S. cerevisiae TPK2Δ strain to produce pseudohyphae. P. brasiliensis TPK1-GFP was transformed into the S. cerevisiae TPK2Δ mutant XPY5a/α and then the transformants were streaked on SLAD agar and an individual colony was observed under 20x magnification. The transformant was unable to produce pseudohyphae in response to a limited nitrogen supply. (B) PbTpk1 localizes to distinct sites in the cytoplasm of S. cerevisiae cells. Confocal microscopy of cells expressing PbTpk1-GFP, in which the nucleus was localized by staining with DAPI. (PDF) [file pone.0136866.s007.pdf]

S3 Fig. The transformation of the diploid *S. cerevisiae TPK2Δ* mutant with *P. brasiliensis* Tpk1. (A) *PbTPK1* does not complement the *S. cerevisiae TPK2Δ* strain to produce pseudohyphae. *P. brasiliensis TPK1-GFP* was transformed into the *S. cerevisiae TPK2Δ* mutant XPY5a/α and then the transformants were streaked on SLAD agar and an individual colony was observed under 20x magnification. The transformant was unable to produce pseudohyphae in response to a limited nitrogen supply. **(B) PbTpk1 localizes to distinct sites in the cytoplasm of *S. cerevisiae* cells.** Confocal microscopy of cells expressing PbTpk1-GFP, in which the nucleus was localized by staining with DAPI.

A

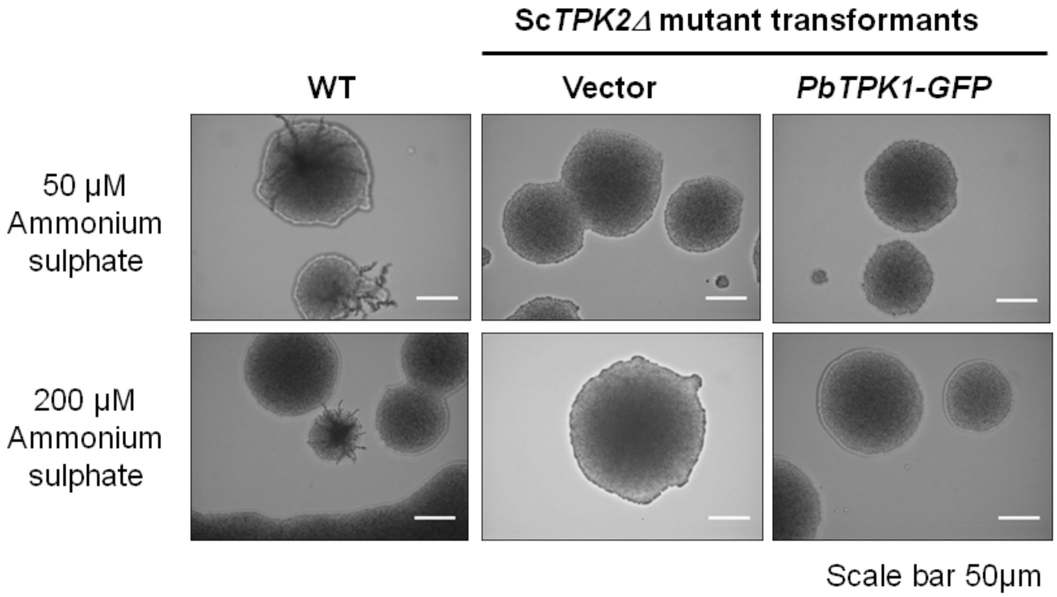

B

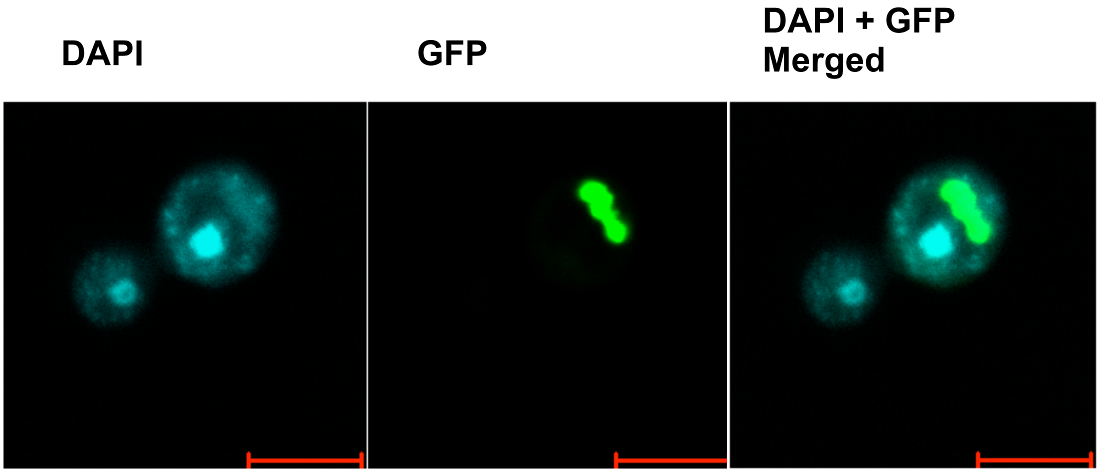

Scale bar 5 µm
