# Supplementary material for: A Gβ protein and the TupA Co-Regulator Bind to Protein Kinase A Tpk2 to Act as Antagonistic Molecular Switches of Fungal Morphological Changes
Source: PLoS One. 2015 Sep 3;10(9):e0136866. doi: 10.1371/journal.pone.0136866 (PMC4559445; doi:10.1371/journal.pone.0136866)

S4 Fig. PbGpa1 does not block pseudohyphae formation. (A) *P. brasiliensis GPA1-GFP* was transformed with or without *PbTPK2* into the *S. cerevisiae* MLY61a/α (WT) and the *TPK2Δ* mutant XPY5a/α (XPY) and then the transformants were streaked on SLAD agar and an individual colony was observed under 20x magnification. The XPY5a/α/*PbGPA1* transformant was unable to produce pseudohyphae but the MLY61a/α/*PbGPA1* transformant and the XPY5a/α/*PbTPK2/PbGPA1* co-transformant could both produce pseudohyphae in response to a limited nitrogen supply. PbGpa1 does not block bind Tpk2 to block pseudohyphae formation. **(B) Pan-cellular distribution of PbGpa1 in *S. cerevisiae* cells.** Confocal microscopy of cells expressing PbGpa1-GFP, in which the nucleus was localized by staining with DAPI.

A

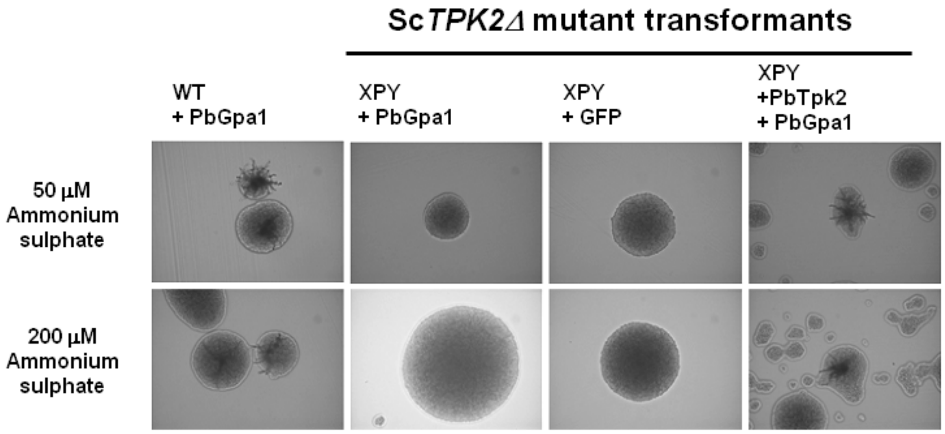

B

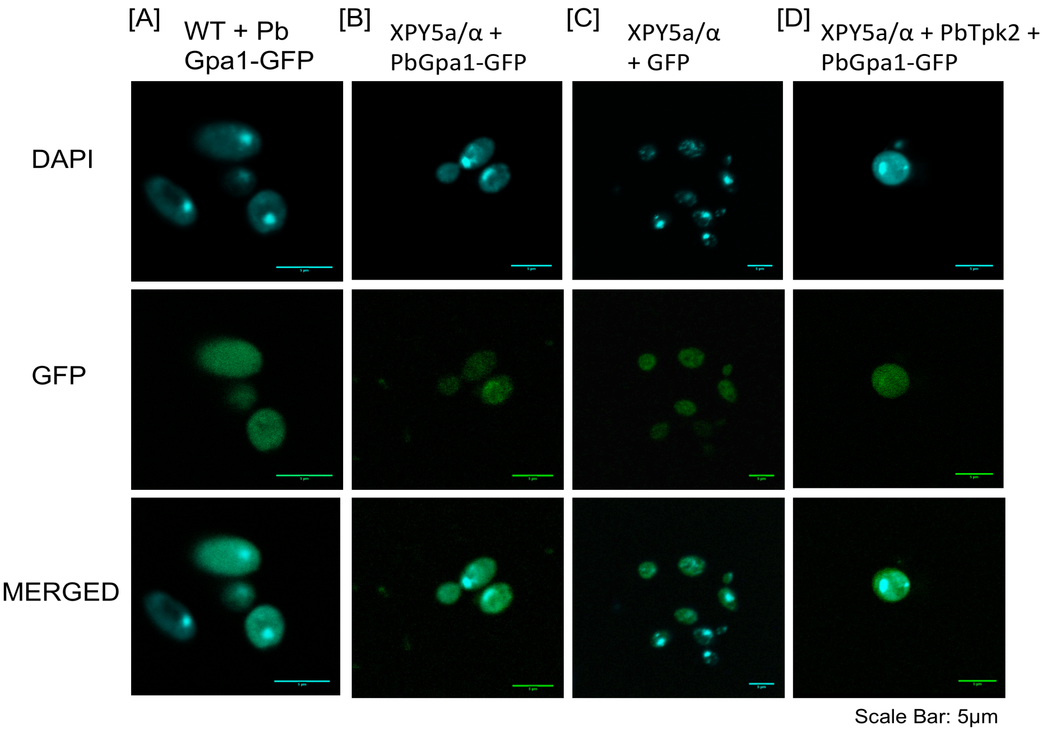

Supplement: S4 Fig — (A) P. brasiliensis GPA1-GFP was transformed with or without PbTPK2 into the S. cerevisiae MLY61a/α (WT) and the TPK2Δ mutant XPY5a/α and then the transformants were streaked on SLAD agar and an individual colony was observed under 20x magnification. The XPY5a/α/PbGPA1 transformant was unable to produce pseudohyphae but the MLY61a/α/PbGPA1 transformant and the XPY5a/α/PbTPK2/PbGPA1 co-transformant could both produce pseudohyphae in response to a limited nitrogen supply. PbGpa1 does not bind Tpk2 to block pseudohyphae formation. (B) Pan-cellular distribution of PbGpa1 in S. cerevisiae cells. Confocal microscopy of cells expressing PbGPA1-GFP, in which the nucleus was localized by staining with DAPI. (PDF) [file pone.0136866.s008.pdf]
