# Supplementary material for: A Gβ protein and the TupA Co-Regulator Bind to Protein Kinase A Tpk2 to Act as Antagonistic Molecular Switches of Fungal Morphological Changes
Source: PLoS One. 2015 Sep 3;10(9):e0136866. doi: 10.1371/journal.pone.0136866 (PMC4559445; doi:10.1371/journal.pone.0136866)
Supplement: S5 Fig — Alignment was done with Vector NTI 6.0. GenBank protein accession numbers are indicated as following the name of proteins. The predicted N-terminal coiled-coil domain and seven WD40 repeats are shown with black lines. (PDF) [file pone.0136866.s009.pdf]

S5 Fig. PbTupA is highly conserved with TupA from *P. marneffei* and *A. nidulans*. Alignment was done with Vector NTI 6.0. GenBank protein accession numbers are indicated as following the name of proteins. The predicted N-terminal coiled-coil domain and seven WD40 repeats are shown with black lines.

```
                                                    coiled-coil
                     (1) 1         10        20        30        40        50        60        70
          PbTupA     (1) --------------------------MYNPHRGMVTAPNSRLTELLDQLRQEYENQSRNTGEFEHQLTGQ
 PmTupA-AAL99251     (1) --------------------------MYNAHRGMVPAPNNRLTELLEQLRQEFESQSRSTGEFEHQLTGQ
 AnTupA-AAG28504     (1) MRSIDQPLLTRLPSSGRAQTSQLPLTMYNAHRGMVPAPNSRLNELLDQLRQEFENQSRSTGEFEHQLTGQ
       Consensus     (1)                           MYNAHRGMVPAPNSRLTELLDQLRQEFENQSRSTGEFEHQLTGQ
                    (71) 71        80        90        100       110       120       130       140
          PbTupA    (45) LQEMEMIRQKVYQLEQAQIKMKQEYEAEIRTLRMELESRGVHLPQSHIGGPPTHAGPSQAPPPALGHGPA
 PmTupA-AAL99251    (45) IQEMEMIRQKVYQLEQAQIKIKAEYENEIRVLRHELESRGVQTVPSHVGGPTHAG-PS-QAPPPALGHGTN
 AnTupA-AAG28504    (71) LQEMEMIRQKVYQLEQAQLKMKQDYEAEIRMLRHELESRGVQPVASHVAVPAQHGGPSQAPPPALGHGPS
       Consensus    (71) LQEMEMIRQKVYQLEQAQIKMKQEYEAEIR LRHELESRGVQ V SHVGGP  HGGPSQAPPPALGHGP
                   (141) 141       150       160       170       180       190       200       210
          PbTupA   (115) NLFGGIMTNPGGGAPALAPPPPPQDQQQPPQHALQQPGSGAQ-GPPQPQQGAFGGYQPASAVNGYGPPPP
 PmTupA-AAL99251   (113) NLFGGIMANQGGSGPGLAPPPP-QEQQ-PPQHGLQQPAPAAQQGPPQPPQSSYGGYQPSAGVNGYGPPP-
 AnTupA-AAG28504   (141) NLFGGIMANQGGSGPGLAPPPP-QDQQ-PSQHTLQQPAAAAQPGAPQPPQSSFGGYQPWR--CCYGPPP-
       Consensus   (141) NLFGGIMANQGGSGPGLAPPPP QDQQ PPQH LQQPA AAQ GPPQPPQSSFGGYQP   VNGYGPPP
                   (211) 211       220       230       240       250       260       270       280
          PbTupA   (184) APTSSPGPGKGRASRAPPGPATPQQNHQVAYPDTRASPQMPRPTPPG-QQLSYRVGNTLADLDPDKLPPS
 PmTupA-AAL99251   (180) PPTASPGPGKG-RGRAPPGPATPQQTHQLNYPAPGASPQIPRPTPTG-QLVRERPGNTLANWNPDDLPAS
 AnTupA-AAG28504   (206) PPTASPGPGK--RPRAPPGPATPQQAHQLAYPESRVSPQLARPTPPNQPVVRDRPGNMLANWNPEDLPPS
       Consensus   (211) PPTASPGPGKG R RAPPGPATPQQ HQLAYP  RASPQ PRPTPPG Q VR RPGNTLANWNPDDLPPS

                                                        WD40
                   (281) 281       290       300       310       320       330       340       350
          PbTupA   (253) QKREGSDWYAVFNPEVPRLLDVDLVHHLVHDSVVCCVRFSNDGKFVATGCNRSAQIFDVQSGQLVTSLQD
 PmTupA-AAL99251   (248) QKREGPDWYAVFNPEVQRVLDVELVHHLVHDSVVCCVRFSRDGKYVATGCNRSAQIFDVATGQNVATLQD
 AnTupA-AAG28504   (274) QKKEGVDWYAVFNPEVQRVLDVELVHHLVHDSVVCCVRFSRDGKYLATGCNRSAQIFDVTLGQNVAVLQD
       Consensus   (281) QKREG DWYAVFNPEVQRVLDVELVHHLVHDSVVCCVRFSRDGKYVATGCNRSAQIFDV   GQNVA LQD

                                   WD40                                WD40
                   (351) 351       360       370       380       390       400       410       420
          PbTupA   (323) DSVLDKEGDLYIRSVCFSPDGRYLATGAEDKQIRVWDIQKRTIKHIFSGHEQDIYSLDFARNGRYIASGS
 PmTupA-AAL99251   (318) ES-VDKDGDLYIRSVCFSPDGKYLATGAEDKQIRVWDIATRSIKHVFTGHEQDIYSLDFAGNGRYIASGS
 AnTupA-AAG28504   (344) ES-VDKSGDLYIRSVCFSPDGKYLATGAEDKQIRVWDIATRTIKHIFSGHEQDIYSLDFAGNGRYIASGS
       Consensus   (351) ES VDK GDLYIRSVCFSPDGKYLATGAEDKQIRVWDIATRTIKHIFSGHEQDIYSLDFAGNGRYIASGS

                                             WD40
                   (421) 421       430       440       450       460       470       480       490
          PbTupA   (393) GDKTVRLWDIVDGKQELILNIEDGVTTVAISPDGRFVAAGSLDKSVRVWDTTTGYLIERLENPDGHMDSV
 PmTupA-AAL99251   (387) GDKTVRLWDVLEGKLVYTLSIEDGVTTVAMSPDGHYVAAGSLDKSVRVWDTTTGYLVERLENPDGHKDSV
 AnTupA-AAG28504   (413) GDKTVRLWDIAEGKLVYTLSIEDGVTTVAMSPDGLYVAAGSLDKTVRVWDTTTGYLVERLENPDGHKDSV
       Consensus   (421) GDKTVRLWDI EGKLVYTLSIEDGVTTVAMSPDG YVAAGSLDKSVRVWDTTTGYLVERLENPDGHKDSV

                             WD40                                      WD40
                   (491) 491       500       510       520       530       540       550       560
          PbTupA   (463) YSVAFAPNGRDLVSGSLDKTIKLWELTPPRGMVPGSGPKGGKCVRTFEGHKDFVLSVCLTPDGRWVMSGS
 PmTupA-AAL99251   (457) YSVAFAPNGRDLVSGSLDKTIKMWELTAPRGMLPGTGVKGGKCVRTFEGHKDFVLSVCLTPDGHWVMSGS
 AnTupA-AAG28504   (483) YSVAFAPNGKELVSGSLDKTIKLWELNLPRQQYNSAGK-GGKCHRTFEGHKDFVLSVCLTPDGHWVMSGS
       Consensus   (491) YSVAFAPNGRDLVSGSLDKTIKLWELT PRGM PG G KGGKCVRTFEGHKDFVLSVCLTPDGHWVMSGS

                                               WD40
                   (561) 561       570       580       590       600       610             628
          PbTupA   (533) KDRGVQFWDPATGNAQMMLQGHKNSVISVAPSPVGNLFATGSGDMRARIWSYSNWDPRRS--------
 PmTupA-AAL99251   (527) KDRGVQFWDPNTGSAQMMLQGHKNSVISVAPSPTNNLFATGSGDMRARIWRYVEFPE-----------
 AnTupA-AAG28504   (552) KDRGVQFWDPITGNAQMMLQGHKNSVISVAPSPTGNLFATGSGDMRARIWRYASFPNPISLPKIWMLG
       Consensus   (561) KDRGVQFWDP TGNAQMMLQGHKNSVISVAPSPTGNLFATGSGDMRARIWRY  FP   S
```
