# Supplementary material for: A Gβ protein and the TupA Co-Regulator Bind to Protein Kinase A Tpk2 to Act as Antagonistic Molecular Switches of Fungal Morphological Changes
Source: PLoS One. 2015 Sep 3;10(9):e0136866. doi: 10.1371/journal.pone.0136866 (PMC4559445; doi:10.1371/journal.pone.0136866)
Supplement: S6 Fig — The S. cerevisiae diploid strain MLY61a/α (WT) and its TPK2Δ mutant XPY5a/α were transformed with the PbTUPA, ScTUP1, PbTPK2 and PbGPB1 as indicated. To allow selection, generally, constructs for the expression of the PbTupA-mRFP, ScTup1-mRFP, PbTpk2-FL-mRFP and PbGpb1-GFP, PbTpk2(226–583)-GFP, PbTpk2(226–583)-GFP K301R and PbTpk2(265–583)-GFP fusion proteins were used and the transformants, selected on the basis of their fluorescence The cells were analysed for pseudohyphal growth in SLAD agar containing 50 μM (upper panel) or 200 μM (middle panel) ammonium sulphate, and for invasive growth into SD—ura agar medium (bottom panel). Single colonies from the agar plate were observed at 20x magnification in an Eclipse E-400 microscope (upper and middle panels; scale bar 50 μm) and in a Leica M165 FC stereo fluorescence microscope (bottom panel; scale bar 0.8 inches). WT cells expressing PbTupA were hyperfilamentous; whilst those expressing ScTup1 did not produce pseudohyphae. XPY cells expressing PbTupA produced few pseudohyphae; whilst those expressing PbTupA with PbTpk2, but not a kinase defective K301R derivative, were hyperfilamentous, indicating the requirement for a functional PKA. The co-expression of PbGpb1 with PbTupA repressed the filamentous growth of the XPY/PbTPK2 but not the WT cells, indicating that PbGpb1 specifically inhibits PbTpk2. (PDF) [file pone.0136866.s010.pdf]

S6 Fig. PbTupA induces hyperfilamentous growth that can be repressed by PbGpb1. The *S. cerevisiae* diploid strain MLY61a/α (WT) and its *TPK2Δ* mutant XPY5a/α (XPY) were transformed with the *PbTUPA, ScTUP1, PbTPK2* and *PbGPB1* as indicated. To allow selection, generally, constructs for the expression of the PbTupA-mRFP, ScTup1-mRFP, PbTpk2-GFP and PbGpb1-GFP fusion proteins were used and the transformants, selected on the basis of their green and/or red fluorescence. The cells were analysed for pseudohyphal growth in SLAD agar containing 50 μM (upper panel) or 200 μM (middle panel) ammonium sulphate, and for invasive growth into SD –ura agar medium (bottom panel). Single colonies from the agar plate were observed at 20x magnification in an Eclipse E-400 microscope (upper and middle panels; scale bar 50 μm) and in a Leica M165 FC stereo fluorescence microscope (bottom panel; scale bar 0.8 inches). WT cells expressing PbTupA were hyperfilamentous; whilst those expressing ScTup1 did not produce pseudohyphae. XPY cells expressing PbTupA produced few pseudohyphae; whilst those expressing PbTupA with PbTpk2, but not a kinase defective K301R derivative, were hyperfilamentous, indicating the requirement for a functional PKA. The co-expression of PbGpb1 with PbTupA repressed the filamentous growth of the XPY/*PbTPK2* but not the WT cells, indicating that PbGpb1 specifically inhibits PbTpk2.

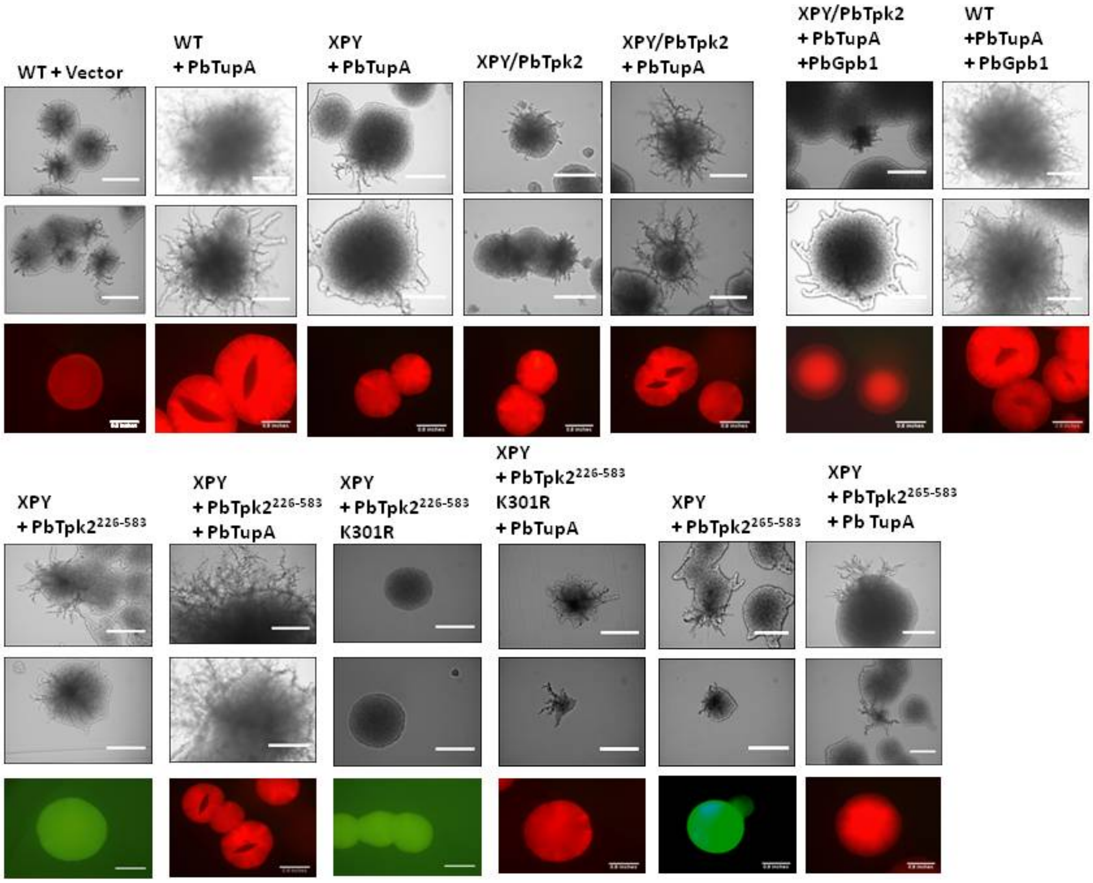

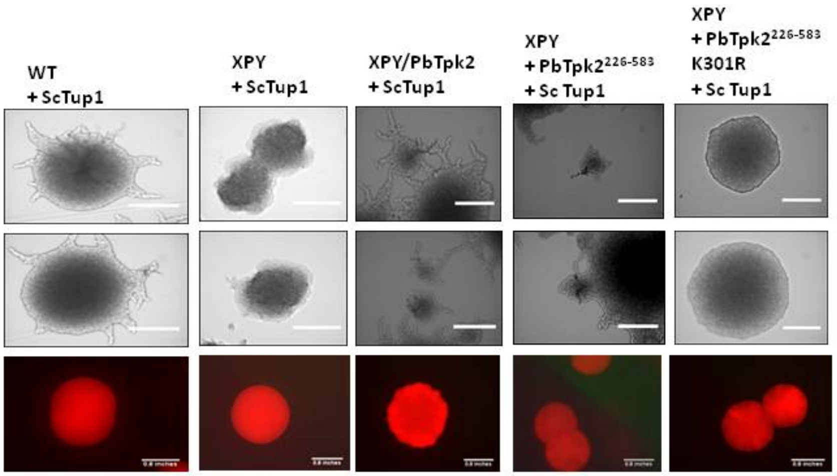
WT
+ ScTup1
XPY
+ ScTup1
XPY/PbTpk2
+ ScTup1
XPY
+ PbTpk2$^{226-583}$
+ Sc Tup1
XPY
+ PbTpk2$^{226-583}$
K301R
+ Sc Tup1
