# Supplementary material for: A Gβ protein and the TupA Co-Regulator Bind to Protein Kinase A Tpk2 to Act as Antagonistic Molecular Switches of Fungal Morphological Changes
Source: PLoS One. 2015 Sep 3;10(9):e0136866. doi: 10.1371/journal.pone.0136866 (PMC4559445; doi:10.1371/journal.pone.0136866)

S7 Fig. PbTupA is localized in the nucleus. *P. brasiliensis TUPA-mRFP* was transformed into the *S. cerevisiae* MLY61a/α (WT) diploid strain and its *TPK2Δ* mutant XPY5a/α and then the transformants were streaked on SLAD agar and an individual colony was observed under 20x magnification. Confocal microscopy of cells expressing PbTupA-mRFP, in which the nucleus was localized by staining with DAPI.

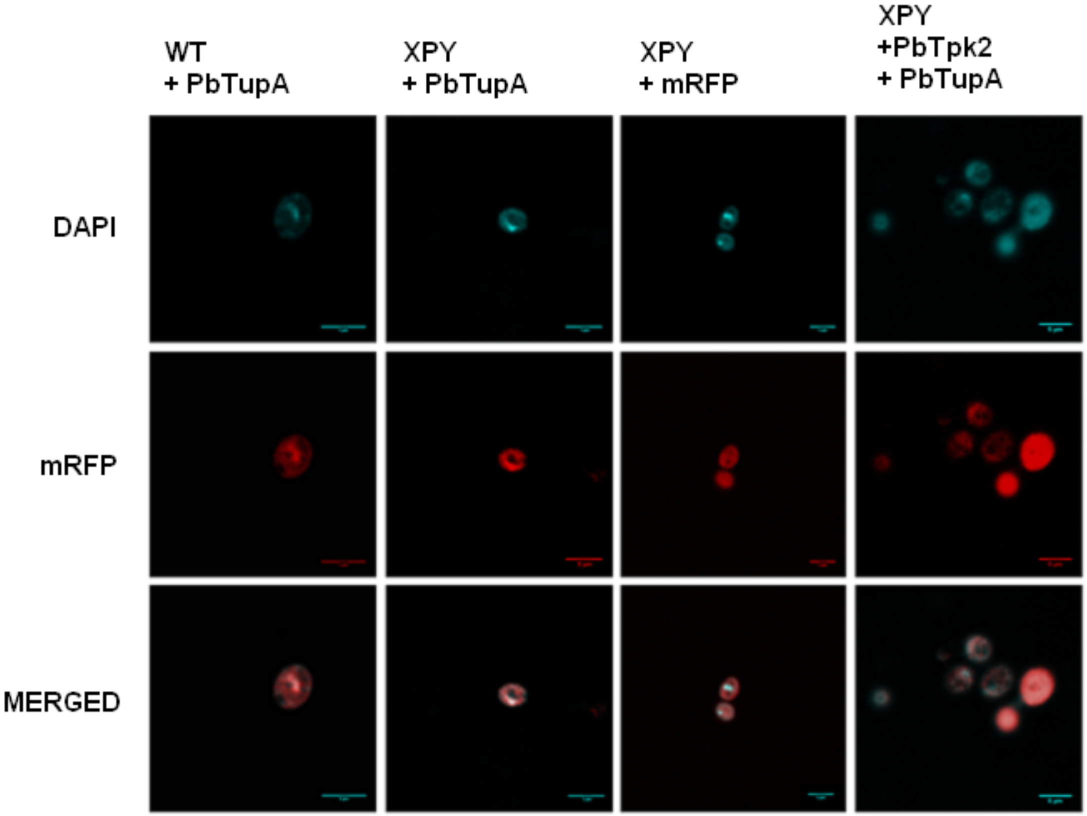

Supplement: S7 Fig — P. brasiliensis TUPA-mRFP was transformed into the S. cerevisiae MLY61a/α (WT) diploid strain and its TPK2Δ mutant XPY5a/α and then the transformants were streaked on SLAD agar and an individual colony was observed under 20x magnification. Confocal microscopy of cells expressing PbTupA-mRFP, in which the nucleus was localized by staining with DAPI. (PDF) [file pone.0136866.s011.pdf]
